# Fibrillarin Contributes to the Oncogenic Characteristics of Colorectal Cancer Cells and Reduces Sensitivity to 5-Fluorouracil

**DOI:** 10.3390/cancers17243900

**Published:** 2025-12-05

**Authors:** Ting Wu, Mounira Chalabi-Dchar, Wei Xiong, Lucie Arnould, Eliezer Aimontche, Sabine Beaumel, Charles Dumontet, Virginie Marcel, Tanguy Fenouil, Jean-Jacques Diaz, Marie Alexandra Albaret, Hichem Claude Mertani

**Affiliations:** 1 LabEx Dev2CAN, Institut Convergence Plascan, Centre de Recherche en Cancérologie de Lyon, Inserm U1052, CNRS UMR5286, Université de Lyon, Université Claude Bernard Lyon, Centre Léon Bérard, CEDEX 08 Lyon, Francecharles.dumontet@chu-lyon.fr (C.D.);; 2 Institute for Cancer Medicine and School of Basic Medical Sciences, Southwest Medical University, Luzhou 646000, China; 3 Laboratoire de Biologie Médicale et d’Anatomie Pathologique, Hospices Civils de Lyon, 69500 Bron, France

**Keywords:** fibrillarin, colorectal cancer, metastasis, ribosome biogenesis

## Abstract

Background/Objectives: Fibrillarin (FBL) is a key nucleolar methyltransferase involved in ribosome biogenesis through 2′-O-ribose methylation of rRNA. While its oncogenic role has been reported in several cancer types, its expression and function in human colorectal cancer (CRC) have remained largely unexplored. This study aims to investigate the expression of FBL in human CRC tissues and cell lines and to determine its functional role in tumor progression and metastasis. Methods: We examined FBL expression in paired human CRC primary tumors and liver metastases using immunohistochemistry. Functional studies were performed using SW-480 (primary tumor) and SW-620 (lymph node metastasis) CRC cell lines derived from the same patient. Cell migration, invasion, and 3D spheroid growth were analyzed following FBL downregulation. In vivo tumor growth was assessed in SCID mice xenografted with FBL-deficient cells. Molecular changes were explored through phosphorylation arrays and Western blotting. Results: FBL expression was significantly higher in human metastatic lesions than in primary tumors. FBL downregulation impaired migration, invasion, and spheroid growth in SW-480 and SW-620 cells and reduced tumor growth in vivo. Mechanistically, FBL inhibition decreased activation of MAPK/ERK, PI3K/AKT, and JNK/p38 pathways and reduced phosphorylation of the transcription factor CREB. Conclusions: Our study identifies FBL as a potential contributor to colorectal cancer progression, with elevated expression associated particularly with metastatic disease. By demonstrating that FBL expression is elevated in patient-derived metastatic tissues and functionally promotes migration, invasion, and tumor growth, our findings expand the role of ribosome biogenesis factors beyond protein synthesis. The observed suppression of key oncogenic pathways and CREB phosphorylation upon FBL inhibition suggests that FBL integrates ribosomal regulation with cancer cell signaling. These insights open new avenues for targeting nucleolar activity in advanced CRC and highlight FBL as a potential biomarker and therapeutic target in metastatic disease.

## Simple Summary

This study investigates the role of fibrillarin, a nucleolar methyltransferase essential for ribosome biogenesis, in CRC progression and metastasis. While fibrillarin’s role in ribosomal RNA modification is well established, its expression and functional relevance in human colorectal tumors—particularly metastatic lesions—has not been previously characterized. Using paired CRC cell lines derived from the same patient (primary tumor: SW-480; lymph node metastasis: SW-620), we demonstrate that fibrillarin supports tumor cell migration, invasion, and growth in both 2D and 3D models. In vivo, fibrillarin inhibition significantly reduced tumor growth in SCID mouse xenografts. Importantly, immunohistochemical analysis of human CRC biopsies revealed that fibrillarin expression is consistently higher in liver metastases than in matched primary tumors, supporting its role in metastatic adaptation. Mechanistically, our data suggest that fibrillarin may contribute to malignancy by modulating MAPK/ERK and PI3K/AKT pathways and regulating the activity of the transcription factor CREB. These findings identify fibrillarin as a potential driver of CRC progression and metastasis, with possible implications for therapeutic targeting.

## 1. Introduction

Colorectal cancer (CRC) is one of the most prevalent malignancies, with an alarming rise in younger populations [1,2]. While the 5-year survival rate is 64%, it falls below 15% once metastasis occurs [3]. CRC characteristics vary based on tumor location, age, and genetic alterations [4]. Current treatments—surgery, chemotherapy, radiotherapy, and targeted therapies against EGFR and VEGF [4,5]—have improved patient outcomes, but challenges like drug resistance and toxicity persist, making alternative strategies essential [6].

Ribosome biogenesis is emerging as a crucial factor in cancer progression [7,8]. This highly coordinated process involves assembling ~80 proteins with four rRNA molecules, requiring modifications such as 2′-O-ribose methylation by fibrillarin (FBL) [9,10]. FBL, a key nucleolar enzyme, associates with ribosomal proteins Nop56, Nop58, and 15.5K to facilitate proper ribosome formation [11]. While ribosome biogenesis is fundamental to all cells, dysregulated rRNA methylation has been linked to cancer development [12]. FBL is frequently overexpressed in cancers, including breast [13,14], pancreas [15], lung [16], colon [16], prostate [15], liver [17], and acute myeloid leukaemia (AML) [18]. Unlike genetic mutations, its overexpression is largely driven by promoter methylation [17]. In breast cancer, FBL fuels MYC-driven snoRNA biogenesis, and its inhibition induces nucleolar stress and p53 activation [13]. Similarly, in AML, FBL supports MYC translation and ribosome biogenesis, with targeted inhibition via CGX-635 reducing cancer cell survival [18]. In prostate cancer, FBL enhances the translation of oncogenic mRNAs like IGF-IR, further driving tumor growth [15]. Beyond ribosome biogenesis, FBL influences CRC progression by regulating stress response pathways and maintaining nucleolar stability [19]. It facilitates translation of key stress-related proteins, enabling CRC cells to survive endoplasmic reticulum (ER) stress conditions in DLD-1 and SW-480 cells [19]. FBL also collaborates with YBX1 to enhance BRCA1 transcription, boosting DNA repair and resistance to therapy in breast and CRC cells [16]. In endometrial cancer, the SNORD104/FBL complex enhances PARP1 mRNA translation to drive tumor progression, while FBL silencing disrupts this process, leading to tumor growth inhibition [20]. While the role of FBL in cancer is well documented, its involvement in metastasis is less understood. In hepatocellular carcinoma (HCC), high FBL, MYC, and E2F levels are linked to lung metastases and poor prognosis [21]. Here, we explored the role of FBL in CRC using primary SW-480 and metastatic SW-620 cell lines, as well as patient biopsies. FBL inhibition reduced migration, invasion, and tumor growth, particularly in SW-480 cells, and altered epithelial–mesenchymal transition markers. Tumor analysis revealed even higher FBL expression in metastases than in primary tumors, highlighting its role in metastatic adaptation and survival.

## 2. Materials and Methods

### 2.1. Cell Culture

Colorectal cancer cell lines SW-480, SW-620, HCT116, HCT116-/- (p53 mut) and HT29 were originally purchased from ATCC (Manassas, VA, USA), and human colonic epithelial cells HCEC-1CT from Evercyte (Vienna, Austria). Details are provided in Supplementary Materials and Table S1.

### 2.2. Lentiviral Vectors and Infection

Lentiviral transduction was performed using the pTRIPZ vector system (Thermo Fisher Scientific, Illkirch, France), which includes a tetracycline-inducible promoter (TRE), a TurboRFP fluorescent reporter, and a puromycin resistance gene (IRES-Puro) for selection. This vector expresses a short hairpin RNA in a microRNA backbone (shFBLRNAmir), allowing efficient and regulated silencing of FBL. Three stable cell populations were created: a control cell line expressing a non-targeting shRNA (shNS), and two independent FBL-targeting shRNA lines (shFBL1 and shFBL2). See Table S5 for the sequences used. Lentiviral particles were produced in 293T cells and used to transduce SW-480 and SW-620 cells under biosafety level 2 conditions. Following transduction, cells were selected with puromycin (Sigma-Aldrich, Saint-Quentin-Fallavier, France) and maintained under standard culture conditions (37 °C, 5% CO_2_). Expression of shRNAs was induced by treating cells with 1 µg/mL doxycycline (Sigma-Aldrich, Saint-Quentin-Fallavier, France) for four days. In the absence of doxycycline, the cells are referred to as control-shNS, control-shFBL1, and control-shFBL2, representing the baseline (non-induced) state where FBL expression remains unaffected. Upon doxycycline treatment, the TRE promoter drives expression of the shRNA and TurboRFP, enabling both silencing of FBL and visual tracking of induction. Importantly, doxycycline treatment had no effect on FBL expression in the shNS cells, confirming the specificity of the knockdown.

### 2.3. Cell Morphology and Cytoskeleton Analysis

Cells (1.5 × 10^4^) were seeded on 35 mm µ-dish (Ibidi, Gräfelfing, Germany) with rat tail collagen I (Thermo Fisher, Illkirch, France) at 50 µg/mL. The cells were observed and photographed using the Zeiss Observer and Zeiss confocal (880 NLO) (Zeiss, Jena, Germany). Phalloidin-conjugated TRITC was used to visualize the actin filaments of the cytoskeleton.

### 2.4. Protein Extraction and Western Blotting

Immunoblotting was performed as previously described [13] and detailed methods are provided in Supplementary Materials and Table S2.

### 2.5. Real-Time Quantitative PCR Analysis

RT-qPCR g was performed as previously described [13] and detailed methods are provided in Supplementary Materials and Table S3.

### 2.6. Immunofluorescence

Immunofluorescence was performed as previously described [13] and detailed methods are provided in Supplementary Materials and Table S4.

### 2.7. Cell Growth, Viability, and Apoptosis Assays

Cell essays were performed as previously described [13] and detailed methods are provided in Supplementary Materials.

### 2.8. Colony Formation Assay

Colony formation assay was performed as previously described [13] and detailed methods are provided in Supplementary Materials.

### 2.9. Cell Migration and Invasion Assay

Migration and invasion assay were performed as previously described [13] and detailed methods are provided in Supplementary Materials.

### 2.10. Spheroid Growth and Invasion Assays

SW480-shNS, SW480-shFBL1/2, SW620-shNS, and SW620-shFBL1/2 cells were treated with doxycycline for four days. Following this, 3000 cells per well from each condition were plated in 96-well ultra-low attachment plates (Corning, Borre, France) to promote spheroid formation. The cells were cultured for six days in 200 µL of medium and imaged daily using the Incucyte system (Sartorius, Göttingen, Germany) at 37 °C. In some plates, 5-FU (10 µg) was added one day after spheroid formation. Image analysis was conducted using Incucyte 2022B Rev2 software (Sartorius, Göttingen, Germany). For spheroid invasion analysis, the spheroids formed after six days were carefully transferred into a liquid Matrigel© (Corning, Amsterdam, The Netherlands) solution (diluted 1:50) and allowed to solidify at 37 °C. Doxycycline was then added on the embedded spheroids to re-induce FBL inhibition and to visualize RFP labeling within the spheroid cells. Photographs were taken daily for six days following the induction of FBL inhibition.

### 2.11. Mice Xenograft Model

Animal work was performed as previously described [22] and detailed methods are provided in Supplementary Materials.

### 2.12. Histopathology and Immunohistochemisty CRC Specimens

Human biological samples and data were obtained from the Tissue-Tumorothèque Est Biobank (CRB-HCL Hospices Civils de Lyon BB-0033-00046) with authorization from the French Ministry of Research (AC-2019-3465). The cohort included 60 tumors—30 primary and 30 metastatic—from 32 patients, with 28 providing both tumor types included. Key clinical characteristics of the patients analyzed are described in Supplementary Figure S1. Formalin-fixed paraffin-embedded blocks were sectioned (4 µm) and stained. IHC was performed using a Ventana BenchMark ULTRA^®^ system (Ventana-Roche Diagnostics, Meylan, France) and an UltraView DAB Detection Kit with an FBL antibody (1:2000, #5821, Abcam, Amsterdam, The Netherlands). IHC slides were digitized using a Leica Biosystems Aperio AT2 scanner (Nussloch, Germany), and signal quantification was performed with QuPath (v0.5.1). For each slide, three tumor regions of interest (ROI) were analyzed and DAB mean optical density was measured across three technical replicates and normalized to FBL expression in normal tissue.

### 2.13. Signaling Pathway Analysis

Phosphorylated proteins extracted from SW-480 shNS and shFBL cells were identified and quantitatively analyzed using the RayBio^®^ C-Series Human MAPK Pathway Phosphorylation Array C1 (Norcross, GA, USA). Total protein extracts from shNS and shFBL cells were prepared using the buffer provided in the kit. These extracts were separately incubated on membranes pre-spotted with 17 human antibodies, each specific to the phosphorylated forms of key signaling proteins involved in various pathways: the PI3K/AKT/mTOR pathway (AKT, mTOR, P70S6K, GSKα, GSK3β), the MAPK/ERK pathway (ERK1/2, MEK, RSK1, RSK2, MSK2), the JNK/p38 pathway (JNK, p38, MKK6), as well as proteins involved in transcriptional regulation (CREB, HSP27) and tumor suppression (P53). Following a one-hour incubation, detection was carried out using a secondary antibody cocktail conjugated to horse radish peroxidase (HRP), targeting the phosphorylated proteins. The signal was detected with anti-rabbit HRP and chemiluminescence. Each membrane included both negative and positive control spots to normalize variations between different spots. Spot density was quantified using the ChemiDoc™ XRS System with Image Lab™ Software (Bio-Rad, Marnes-la-Coquette, France) following the manufacturer’s guidelines.

### 2.14. Statistics

Statistical analysis was conducted using the GraphPad Prism software (version 6.0.). A two-tailed unpaired Student’s *t*-test and Wilcoxon–Mann–Whitney non-parametric *t* test (2 conditions) were used for comparisons between two-group means. All *p*-values were two tailed, and for all analyses, *p* < 0.05 was considered statistically significance. All experiments were performed on independent biological replicates at least 3 times, and images are representative of several fields observed for each experiment. For statistical analysis of FBL expression in primary and metastatic CRC, we used the Mann–Whitney test for unpaired comparisons and the paired *t*-test for paired comparisons. Statistical analysis of RT-qPCR measurements was performed using the Mann–Whitney test by Statgraphics 3 plus software (Statgraphics Centurion).

## 3. Results

### 3.1. FBL Expression in CRC Cell Lines and in Metastatic Colorectal Carcinoma

**Figure 1:**
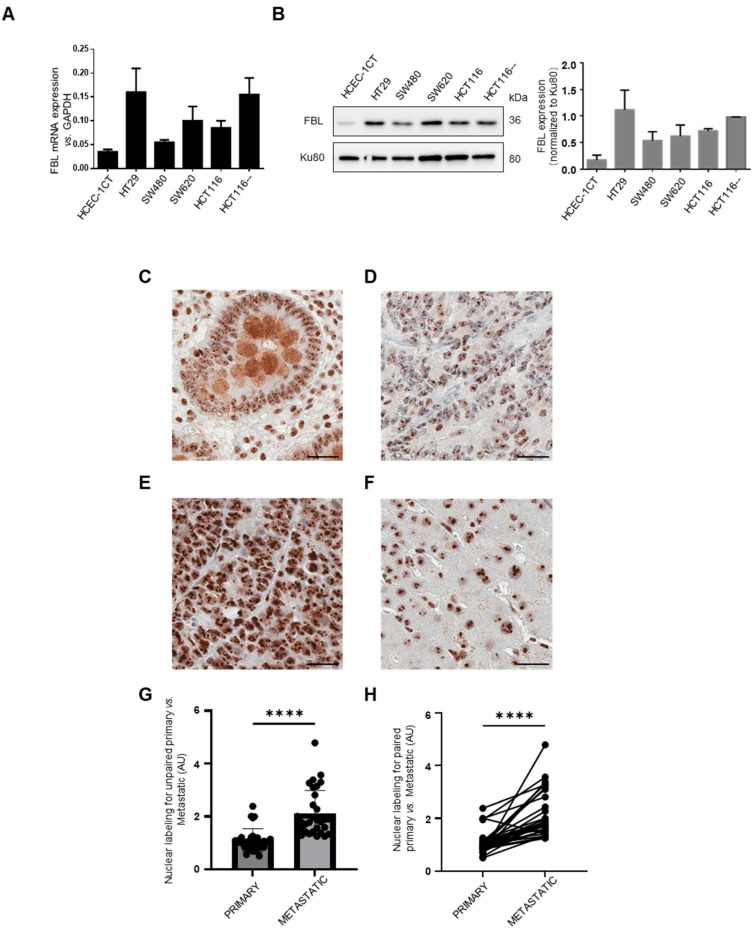
FBL expression in CRC cell lines and immunohistochemical localization and quantification in normal, primary CRC, and paired metastatic CRC tissue. (**A**) Relative FBL mRNA expression levels across various CRC cell lines, including normal HCEC-1CT colon cells, as determined by RT-qPCR. Data are presented as mean ± s.e.m. from independent experiments (*n* = 3). (**B**) (**Left**) Western blot analysis of FBL protein levels in various CRC cell lines compared to normal HCEC-1CT colon cells (The original Western blot images are in Supplementary Material File S1). (**Right**) Quantification of FBL protein expression normalized to Ku80, with individual data points representing biological replicates (mean ± s.e.m., *n* = 3). (**C**–**F**) FBL immunostaining in primary and metastatic CRC sections. The FBL signal was restricted to the nucleolar compartment within both epithelial and stromal cells, in normal adjacent tissue of primary tumours and metastatic lesions (i.e., glandular epithelial intestinal cells (**C**) and hepatocytes (**F**). In primary CRC cells (**D**) FBL was consistently detected, showing strong intensity of labelled nucleoli in all examined cells. Sections from metastatic CRC (**E**) exhibited a significantly higher intensity of the FBL immunohistochemical signal with an increase in the size of labelled nucleoli in all examined cells compared to primary CRC tumours from the same patients. Scale bar = 10 μm. (**G**) Quantitative analysis of unpaired biopsy samples confirmed that FBL signal intensity was significantly higher in liver metastases compared to primary CRC tumour. (**H**) A similar result was observed in matched primary and metastatic biopsy samples from the same patients, with metastases showing significantly higher FBL intensity than primary CRC tumours. For statistical analysis of FBL expression in primary and metastatic CRC. Statistical significance was determined using the Mann–Whitney test for unpaired comparisons and the paired *t*-test for paired comparisons, with *p*-values indicated as **** *p* < 0.001.

We investigated FBL expression in CRC cell lines and normal colonic epithelial cells (HCEC-1CT) using RT-qPCR and Western blotting. FBL was significantly upregulated in all CRC adenocarcinoma cell lines compared to HCEC-1CT (Figure 1A,B). To examine tissue expression, we performed IHC on metastatic CRC patient biopsies. Paired primary and metastatic tumors were analyzed in 28 out of 30 cases. FBL was detected in all 30 primary and metastatic tumors, with strong or moderate signals. FBL immunostaining was examined across normal and cancerous colorectal tissues. In adjacent normal colonic tissue (C), FBL expression was restricted to the nucleolar compartment of both epithelial and stromal cells, with moderate intensity observed in glandular epithelial cells. In primary CRC samples (D), FBL staining was consistently strong, showing pronounced nucleolar labeling in all tumor cells. Metastatic CRC sections (E) displayed a significantly higher FBL signal intensity, accompanied by enlarged nucleoli compared to matched primary tumors from the same patients. In normal liver tissue adjacent to metastases (F), hepatocytes showed only low to moderate nucleolar FBL staining, similar to that seen in normal colon tissue.

Quantitative analysis confirmed higher FBL levels in liver metastases compared to primary CRC tumors in both unpaired and matched samples (Figure 1G,H). No correlation was found between FBL expression and patient age or sex. To investigate potential associations between FBL immunohistochemical staining intensity and clinicopathological parameters, we conducted univariate linear regression analyses. Among all variables tested, only the metastatic versus primary tumor status showed a statistically significant correlation with FBL expression levels. Further analysis using GEPIA (http://gepia.cancer-pku.cn/; accessed on 10 May 2024) of 275 CRC and 349 normal colon/rectal samples confirmed elevated FBL expression in CRC (Figure S1). Kaplan–Meier Plotter (https://kmplot.com/analysis/index.php?p=background, accessed on 1 June 2024) revealed poorer survival and recurrence-free survival in stage IV CRC patients with high FBL levels (Figure S1). These results are further supported by public datasets: UCSC Xena (TCGA-COAD vs. GTEx) shows significantly higher FBL mRNA in tumors than in normal colon, and the Human Protein Atlas reports consistently low FBL expression in healthy tissues with frequent upregulation in cancers. These findings suggest that FBL overexpression is linked to enhanced CRC progression and metastatic potential, and is associated with poorer clinical outcomes.

### 3.2. Inducible FBL Inhibition in Paired Primary SW-480 and Metastatic SW-620 CRC Cells

**Figure 2:**
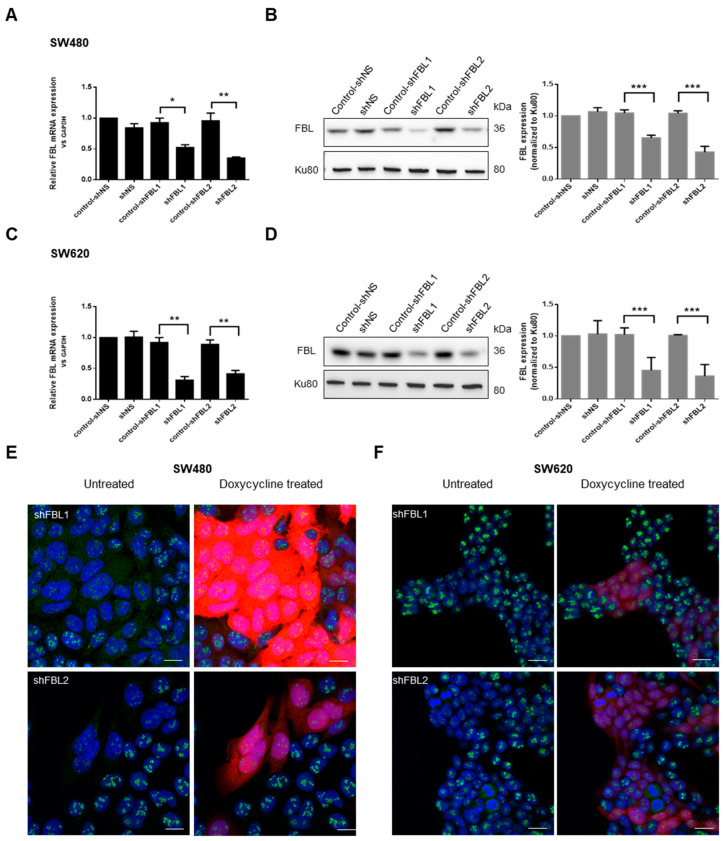
FBL knockdown in paired human CRC cell lines SW-480 and SW-620. (**A**) RT-qPCR validation of FBL knockdown in SW-480 cells following lentiviral infection with doxycycline-inducible shFBL sequences as compared to the control shNS lentiviral vector. (**B**) (**Left**) Western blot analysis of FBL protein levels in SW-480 cells following the knockdown of FBL. (**Right**) Quantification of FBL protein expression normalized to Ku80, with data represented as mean ± s.e.m. from independent experiments (*n* = 5). (**C**) RT-qPCR validation of FBL knockdown in SW-620 cells following lentiviral infection with doxycycline-inducible shFBL sequences as compared to the control shNS lentiviral vector. (**D**) (**Left**) Western blot analysis of FBL protein levels in SW-620 cells following FBL knockdown. (**Right**) Quantification of FBL protein expression normalized to Ku80, with data presented as mean ± s.e.m. from independent experiments (*n* = 5) (The original Western blot images are in Supplementary Material File S1). (**E**) Immunofluorescence analysis in SW-480 cells showing nucleolar localization of FBL and the efficiency of FBL silencing upon doxycycline induction. RFP expression indicates successful transduction, with red fluorescence correlating with the extent of FBL silencing. Nuclei are labelled in blue, FBL in green, and RFP in red. Scale bar = 10 µm. (**F**) Immunofluorescence analysis in SW-620 cells demonstrating nucleolar localization of FBL and successful FBL knockdown following doxycycline treatment. RFP expression correlates with FBL silencing efficiency. Nuclei are labelled in blue, FBL in green, and RFP in red. Scale bar = 10 µm. Images are representative of at least three independent experiments. Statistical significance was determined by unpaired two-tailed *t*-tests, with *p*-values indicated as *p* < 0.05 (*), *p* < 0.01 (**), and *p* < 0.001 (***).

To investigate the role of FBL, we used SW-480 and SW-620 cells as an isogenic model for cancer progression and metastasis. Two short hairpin (sh) RNAs delivered by lentiviral vectors with an RFP reporter were used to stably silence FBL expression, while a non-targeting shNS sequence served as a control. All sequences were doxycycline-inducible. In shNS cell lines, doxycycline had no effect on FBL expression (Figure 2A–D). However, in shFBL1 (−40%) and shFBL2 (−60%) cells, doxycycline significantly reduced FBL mRNA and protein levels (Figure 2A–D). RFP-positive cells showed a marked decrease in nucleolar FBL upon doxycycline treatment (Figure 2E,F).

### 3.3. FBL Regulates Growth and Survival of CRC Cells

To assess FBL inhibition on cell growth, shNS, shFBL1, and shFBL2 cells were cultured in 10% FCS medium, treated with doxycycline for 4 days, and monitored for 6 days using RTCA, MTS assay, and trypan blue exclusion. RTCA was performed using the xCELLigence system (Agilent^®^, Les Ulis, France), which measures changes in electrical impedance as cells adhere and proliferate on microelectrode-coated plates. These impedance changes are translated into a Cell Index (CI), providing dynamic, label-free, and real-time monitoring of cell growth and viability throughout the experiment.

**Figure 3:**
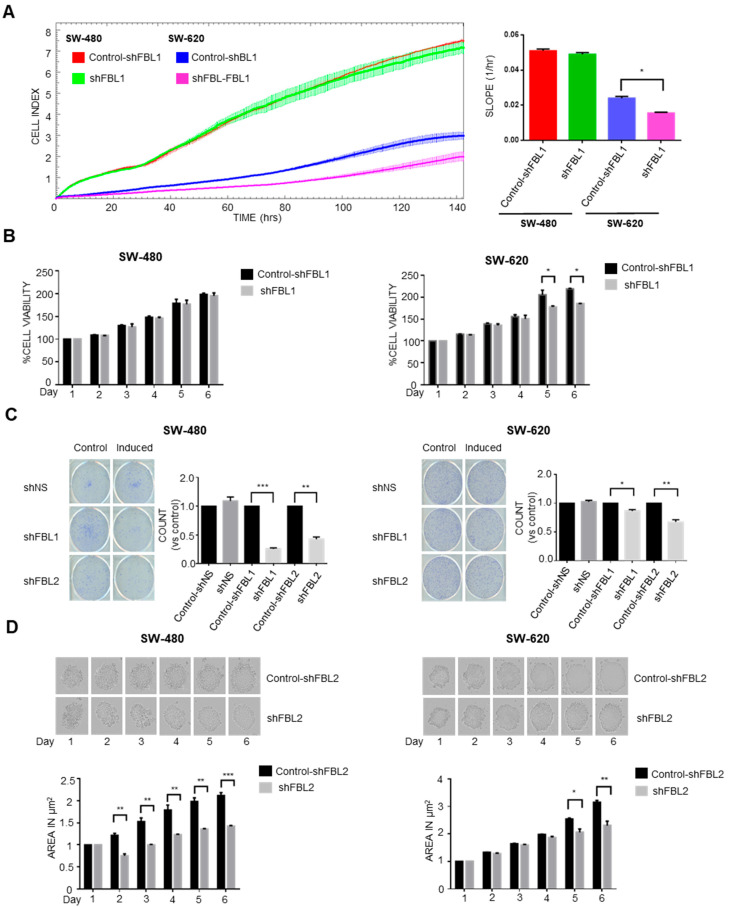
FBL modulates growth and survival of SW-480 and SW-620 CRC cells. (**A**) Real-time cell analysis (RTCA) was used to monitor the growth of SW-480 and SW-620 cells over six days, following doxycycline-induced FBL inhibition. FBL inhibition had no significant impact on the growth of SW-480 cells, while it significantly reduced the growth of SW-620 cells compared to untreated controls. Growth was quantified by the Cell Index slope using GraphPad Prism 6. Data represent the mean ± s.e.m. from four independent experiments. Statistical significance was evaluated using unpaired two-tailed *t*-tests, with *p*-values noted as *p* < 0.05 (*). (**B**) Cell viability of SW-480 (**left**) and SW-620 (**right**) CRC cell lines was assessed using the MTS assay after treatment with or without doxycycline (1 µg/mL). FBL inhibition did not affect the viability of SW-480 cells but significantly reduced the viability of SW-620 cells, with a reduction in cell growth ranging from 10% to 40% at different time points. Data are presented as mean ± s.e.m. from three independent experiments. Statistical analysis was performed using unpaired two-tailed *t*-tests, with significance indicated by *p* < 0.05 (*). (**C**) Colony formation assay was conducted on SW-480 and SW-620 cell lines, treated with or without doxycycline for two weeks. FBL inhibition significantly reduced the number of colonies formed by both SW-480 and SW-620 cells, indicating a decrease in their survival and proliferative capacity. The number of colonies formed was quantified, and data are expressed as mean ± s.e.m. from three independent experiments. Statistical significance was determined using unpaired two-tailed *t*-tests, with *p*-values indicated as *p* < 0.01 (*), *p* < 0.001 (**) and *p* < 0.0001 (***). (**D**) Representative images of spheroids formed by SW-480 (**left**) and SW-620 (**right**) cells embedded in 2% Matrigel, following treatment with or without doxycycline (1 µg/mL) for six days. FBL inhibition significantly reduced the size of spheroids formed by both SW-480 and SW-620 CRC cells. Spheroid areas were measured using ImageJ software 1.54h and normalized to control values. Data represent the mean ± s.e.m. from three independent experiments. Statistical significance was assessed using unpaired two-tailed *t*-tests, with *p*-values indicated as *p* < 0.05 (*), *p* < 0.01 (**), and *p* < 0.001 (***).

FBL inhibition had differential effects on CRC cell growth depending on cell line and assay format. RTCA revealed no change in growth of SW-480 cells, while a significant reduction was observed in SW-620 cells following doxycycline-induced FBL knockdown (Figure 3A). These findings were consistent with MTS assays, which showed sustained growth inhibition in SW-620 cells only of up to 40% (Figure 3B). To assess long-term clonogenic potential, we conducted colony formation assays. Interestingly, FBL inhibition reduced colony numbers in both SW-480 and SW-620 cells, indicating that FBL contributes to cellular fitness over prolonged periods, even in cells that showed minimal effects in short-term proliferation assays (Figure 3C). This unexpected result suggests that the role of FBL in supporting tumor cell growth may be more pronounced under conditions involving prolonged cell–matrix interactions. To further investigate whether this discrepancy could be influenced by microenvironmental context, we used a 3D spheroid model to assess cell-to-cell interactions. In contrast to the limited effect observed in 2D short-term growth assays, FBL silencing led to a marked reduction in spheroid size and compaction in both SW-480 and SW-620 cells (Figure 3D). These results indicate that FBL supports CRC cell growth in both 2D and 3D contexts, with greater effects observed in environments that mimic in vivo architecture.

### 3.4. FBL Regulates CRC Cell Phenotype and Gene Expression of EMT Related Factors

**Figure 4:**
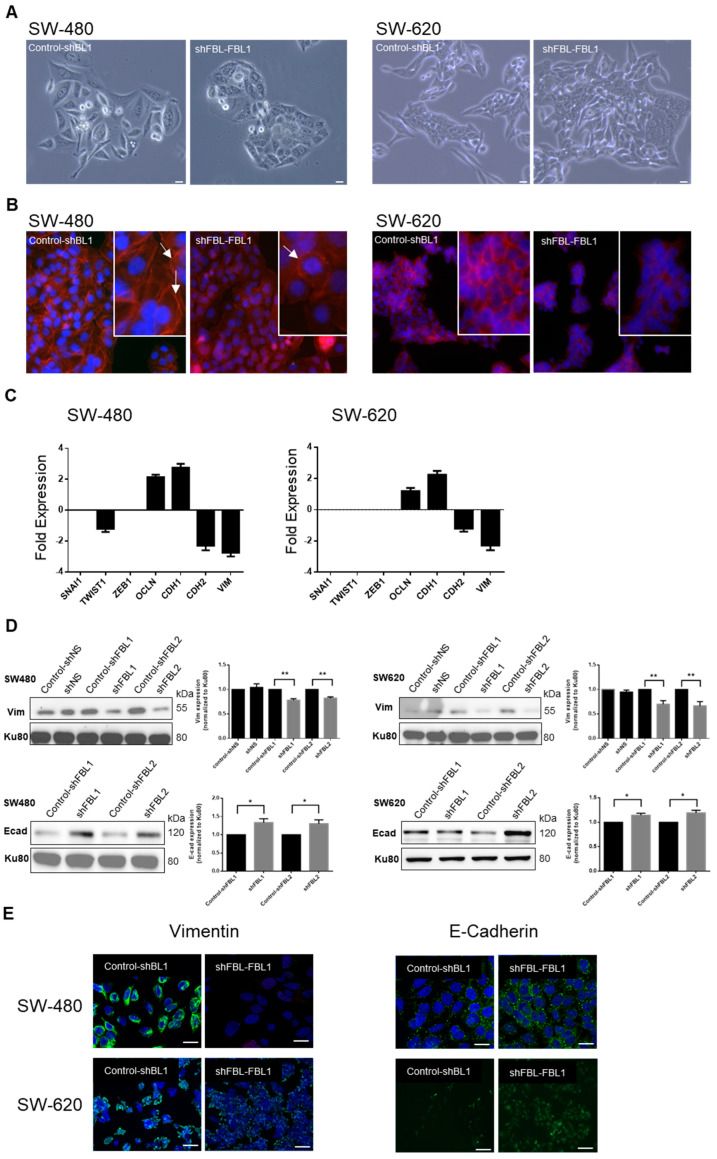
FBL modulates the phenotype and alters expression of EMT-related factors in SW-480 and SW-620 CRC cells. (**A**) Phase contrast microscopy indicating the morphological changes in SW-480 (**left**) and SW-620 (**right**) cell lines following four days of FBL inhibition. FBL silencing resulted in SW-480 cells becoming more tightly packed, less elongated, and exhibiting fewer pseudopodia and cytoplasmic extensions. In SW-620 cells, FBL inhibition led to a more aggregated and less individualized phenotype. Scale bar = 10 μm. Images are representative of three independent experiments. (**B**) Immunofluorescence staining showing the distribution and organization of F-actin in SW-480 (**left**) and SW-620 (**right**) cell lines after FBL inhibition. FBL silencing caused a shift from polarized F-actin distribution at the leading edge in control cells (white arrows) to a more uniform distribution around the cell periphery and at cell-cell junctions (white arrows). F-actin was stained with Phalloidin-TRITC (red), and nuclei were stained with Hoechst (blue). Scale bar = 10 μm. Images are representative of three independent experiments. (**C**) Real-time qPCR quantification of the expression of EMT-related factors in SW-480 (**left**) and SW-620 (**right**) cell lines following FBL inhibition. TWIST1, CDH2 (N-cadherin), and VIM (Vimentin) were significantly downregulated, while CDH1 (E-cadherin) and OCLN (Occludin) were upregulated in FBL-inhibited cells compared to controls. ZEB1 and SNAI1 expression levels remained unchanged. Data are expressed as mean ± s.e.m. from three independent experiments. (**D**) Western blot analysis showing the expression of Vimentin (**top**) and E-Cadherin (**bottom**) protein levels in SW-480 (**left**) and SW-620 (**right**) cell lines following FBL inhibition. FBL silencing led to a significant increase in E-Cadherin levels and a decrease in Vimentin levels in both cell lines. Data are presented as mean ± s.e.m. from four independent experiments. Statistical significance was determined using unpaired *t*-tests, with *p*-values indicated as *p* < 0.05 (*) and *p* < 0.01 (**), (The original Western blot images are in Supplementary Material File S1). (**E**) Immunofluorescence detection of Vimentin (**left**) and E-Cadherin (**right**) expression. Upon FBL inhibition, IF signal of Vimentin decreased, and E-Cadherin levels increased in both SW-480 (**top**) and SW-620 (**bottom**) cell lines. Vimentin and E-Cadherin are detected as green fluorescence, with nuclei stained blue using Hoechst. Scale bar = 10 μm. Images are representative of three independent experiments.

SW-480 cells, derived from a primary adenocarcinoma, exhibit an epithelial-like morphology, while SW-620 cells, from a lymph node metastasis, show a mesenchymal-like phenotype. Phase contrast microscopy revealed that four days of FBL inhibition made SW-480 cells more compact with fewer pseudopodia, while SW-620 cells became less individualized and more aggregated (Figure 4A). F-actin staining showed a shift from a polarized to a uniform distribution at cell junctions upon FBL inhibition, indicating a transition from a migratory to an adherent phenotype (Figure 4B). To further explore these changes, RT-qPCR of EMT-related genes showed significant downregulation of TWIST1, CDH2, and VIM in shFBL cells, while CDH1 and OCLN were upregulated (Figure 4C). ZEB1 and SNAI1 remained unchanged. Western blot analysis revealed that upon FBL inhibition, E-Cadherin increased, and Vimentin decreased in both cell lines, whereas doxycycline treatment alone had no effect (Figure 4D). Immunofluorescence confirmed these results, showing reduced Vimentin and increased E-Cadherin in both cell lines following FBL inhibition (Figure 4E). These findings suggest that FBL suppression shifts SW-480 and SW-620 cells toward a more epithelial phenotype.

### 3.5. FBL Regulates the Behavior of CRC Cells

**Figure 5:**
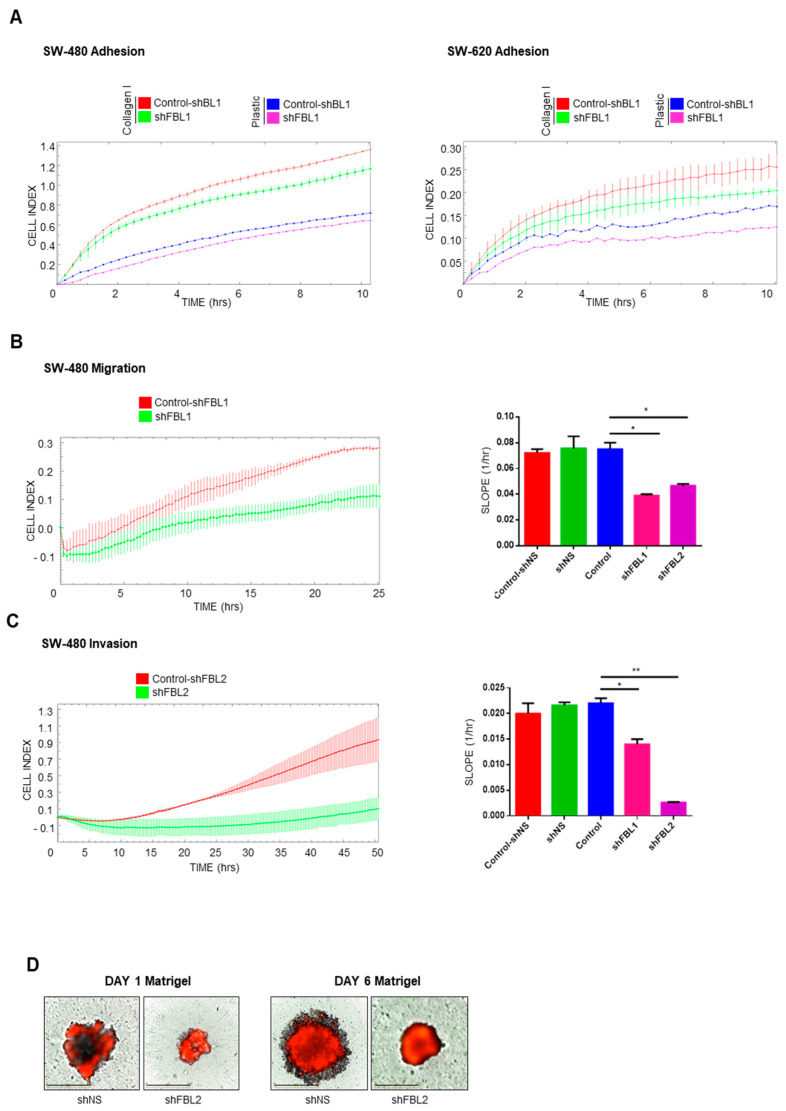
FBL regulates adhesion, migration, and invasion of SW-480 and SW-620 CRC cells. (**A**) RTCA was used to measure cell adhesion dynamics in SW-480 (**left**) and SW-620 (**right**) cell lines following FBL inhibition, both on Collagen I-coated surfaces and uncoated plastic wells. FBL inhibition reduced adhesion in SW-480 cells on both substrates. In SW-620 cells, which exhibit weaker adhesion compared to SW-480, FBL inhibition further decreased cell adhesion, particularly on Collagen I. Data are expressed as mean ± s.e.m. from three independent experiments. (**B**) Migration of SW-480 cells following FBL inhibition was assessed using the RTCA system. FBL inhibition resulted in a significant reduction in cell migration, with a 40–50% decrease in the number of migrating cells compared to controls over a period of 24 h. (**C**) Invasion through Matrigel^®^ (10%) was evaluated using the RTCA system, showing that FBL inhibition substantially delayed invasion, with a 70% reduction in invasive cells over 48 h. (**D**) The invasive potential of SW-480 cells in a 3D environment was analysed using a spheroid Matrigel-invasion assay. Spheroids were pre-treated with doxycycline for four days to induce FBL silencing, then formed in ULA plates for six days. Upon embedding in Matrigel (1/10), FBL silencing resulted in smaller, more compact spheroids, with a lack of RFP-positive cell invasion into the surrounding Matrigel, indicating that FBL is essential for spheroid growth and invasion. Quantification of the Cell Index slope is shown. Data are expressed as mean ± s.e.m. from three independent experiments. Statistical significance was determined using an unpaired *t*-test, with *p*-values indicated as *p* < 0.05 (*) and *p* < 0.01 (**).

The observed changes in cell shape, cytoskeleton, and EMT-related factors suggested alterations in adhesion, migration, and invasion. We assessed adhesion by measuring electrical impedance using the RTCA system. FBL inhibition reduced SW-480 cell adhesion on both collagen I and plastic surfaces (Figure 5A, left). SW-620 cells exhibited weaker adhesion (SW-480, 1.3 vs. SW-620, 0.25 cell index at 10 h), which was improved on a collagen I matrix but decreased upon FBL inhibition (Figure 5A, right). Since FBL inhibition reduced cytoplasmic extensions and adhesion, we examined cell migration. The RTCA system failed to detect SW-620 migration due to their amoeboid-type of motion and weak adhesion. However, SW-480 cells displayed mesenchymal migration, forming pseudopodia and filopodia (Figure 5B, left). FBL inhibition reduced SW-480 migration by 40–50% after 24 h (Figure 5B, right). For invasion analysis, the RTCA assay showed a strong delay in impedance signals upon FBL inhibition in SW-480 cells, indicating reduced invasion (Figure 5C, left). Over two days, we quantified a > 70% decrease in invading cells (Figure 5C, right). We further evaluated the impact of FBL inhibition on 3D invasion using a spheroid-based Matrigel assay. Spheroids were formed from SW-480 cells in ULA plates and treated with doxycycline for four days to induce shRNA expression. These spheroids were then embedded in a Matrigel matrix left to solidified (1:50 dilution) and monitored over time. As shown in Figure 5D (left), FBL silencing reduced both spheroid size and compactness, indicating impaired 3D growth under anchorage-independent conditions. In parallel, invasion into the surrounding matrix was assessed using the RFP reporter signal. Control spheroids (shNS) exhibited RFP-positive cellular extensions breaching the Matrigel boundary, whereas FBL-inhibited spheroids displayed a complete absence of such invasive protrusions (Figure 5D, right). Given the rigidity of the Matrigel, this lack of invasion strongly supports the role of FBL in facilitating active cell penetration into the ECM. This qualitative assay, performed in support of the RTCA Boyden chamber results, provides complementary visual evidence that FBL is required for both 3D tumor growth and matrix invasion in CRC cells.

### 3.6. FBL Inhibition Changes the Sensitivity of CRC Cells to 5-FU

**Figure 6:**
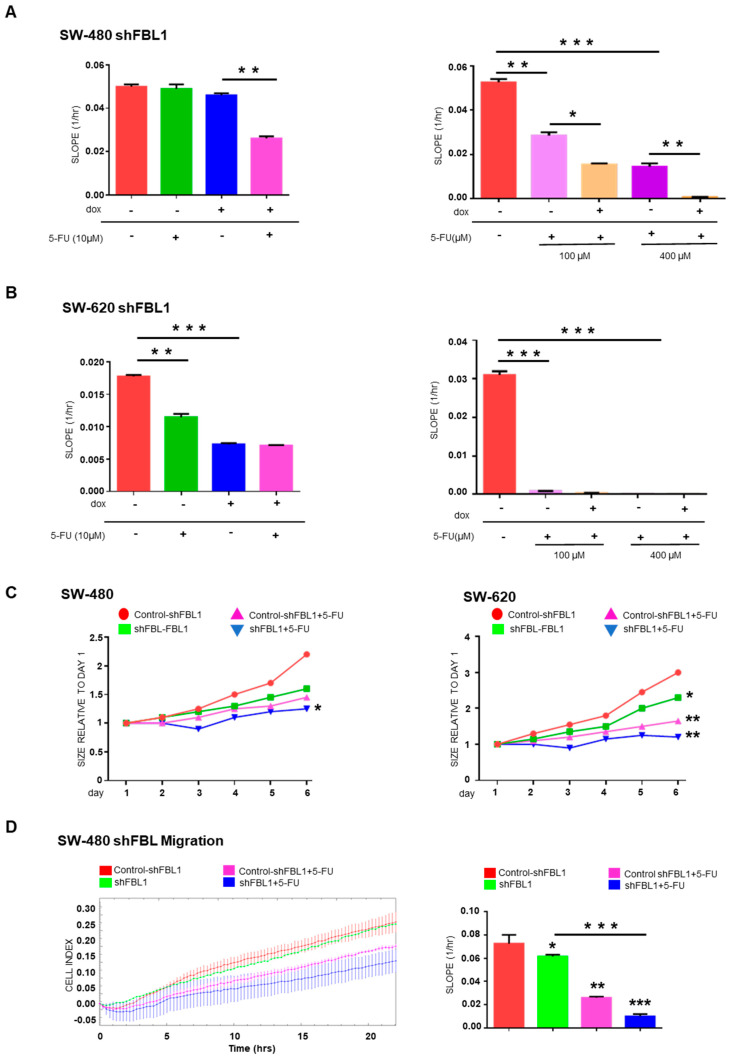
FBL modulates the sensitivity of SW-480 and SW-620 CRC cells to 5-FU. (**A**) RTCA was used to determine the IC50 value of 5-FU in SW-480 (**left**) and SW-620 (**right**) cell lines by measuring cell growth under varying concentrations of 5-FU. SW-480 cells exhibited complete resistance to 5-FU at 10 µM and a dose-dependent cytotoxic response at higher concentrations, with an IC50 of 75 µM. In contrast, SW-620 cells showed significant sensitivity to 5-FU, with an IC50 of 20 µM. Data are expressed as mean ± s.e.m. from three independent experiments. (**B**) RTCA was employed to assess the effect of FBL inhibition on the growth of SW-480 (**left**) and SW-620 (**right**) cells in the presence or absence of 10 µM of 5-FU. FBL inhibition sensitized SW-480 cells to 10 µM 5-FU, leading to reduced cell growth, whereas the primary sensitivity of SW-620 cell to 5-FU was not further enhanced by FBL inhibition. Data are expressed as mean ± s.e.m. from three independent experiments. (**C**) Spheroid formation and growth were monitored in SW-480 (**left**) and SW-620 (**right**) cells embedded in 2% Matrigel^®^ (Corning, Amsterdam, The Netherlands) in the presence of 10 µM 5-FU, using the Incucyte^®^ system. FBL inhibition further reduced the growth of SW-480 spheroids treated with 10 µM 5-FU, indicating increased sensitivity under 3D conditions. Similarly, FBL inhibition enhanced the cytostatic effect of 5-FU in SW-620 spheroids, resulting in a greater reduction in spheroid size compared to controls. Data are expressed as mean relative ratio from three independent experiments. (**D**) RTCA was used to evaluate the effect of 10 µM 5-FU on the migration of SW-480 cells following FBL inhibition. Inhibiting FBL further reduced the migratory capacity of SW-480 cells in response to 5-FU treatment. Quantification of the Cell Index slope is shown, with data expressed as mean ± s.e.m. from three independent experiments. Statistical significance was determined using an unpaired *t*-test, with *p*-values indicated as *p* < 0.05 (*), *p* < 0.01 (**), and *p* < 0.001 (***).

We investigated whether FBL inhibition affects CRC cells response to 5-FU, the standard chemotherapeutic agent used in CRC treatment. In SW-480 cells, neither FBL inhibition nor treatment with low-dose 5-FU (10 µM) alone significantly impacted cell growth over five days (Figure 6A, left). However, their combination led to a significant reduction in cell growth, suggesting an additive effect under these conditions. At higher 5-FU concentrations (100 and 400 µM), a marked cytotoxic effect was observed in SW-480 cells, which was further enhanced by FBL inhibition (Figure 6A, right). In SW-620 cells, which are more sensitive to 5-FU, treatment with 10 µM alone significantly reduced cell growth. FBL inhibition also produced a comparable growth reduction (Figure 6B, left). However, their combination did not yield additional effects, indicating that FBL inhibition alone is sufficient to suppress SW-620 cell growth under these 2D culture conditions. At higher 5-FU doses (100 and 400 µM), cytotoxicity was near complete, thereby masking any additive effect from FBL inhibition (Figure 6B, right). To better mimic the tumor microenvironment, we evaluated drug response in 3D Matrigel (2%)-embedded spheroids. In SW-480 spheroids, 10 µM 5-FU slowed growth modestly, but when combined with FBL inhibition, a clear enhancement of the cytostatic effect was observed (Figure 6C, left). In SW-620 spheroids, 5-FU (10 µM) alone reduced growth by ~40%, while the combination with FBL inhibition resulted in a further decrease, reaching ~50% growth reduction (Figure 6C, right). These data suggest that FBL inhibition sensitizes CRC cells to low-dose 5-FU specifically in 3D culture models, where cellular architecture and microenvironmental cues are better preserved. Finally, we assessed whether FBL inhibition enhances the anti-migratory effects of 5-FU in SW-480 cells. Using RTCA, we found that 10 µM 5-FU alone partially reduced cell migration. FBL inhibition further potentiated this effect, leading to a more pronounced suppression of migratory capacity (Figure 6D).

### 3.7. FBL Alteration of Signaling Pathways in CRC Cells

**Figure 7:**
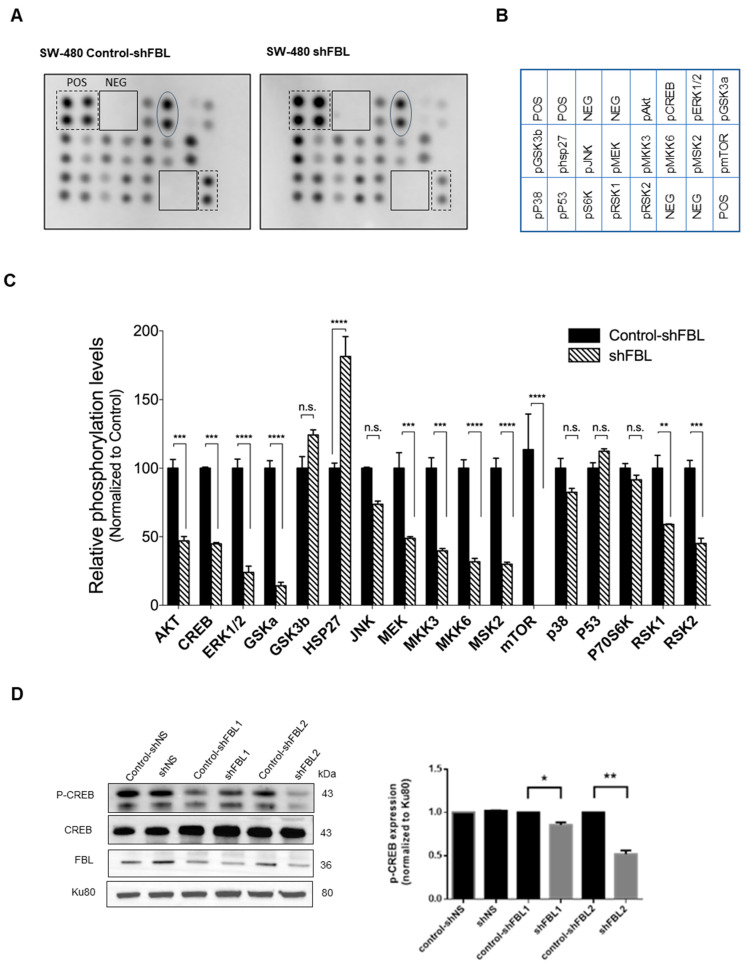
FBL modulates signaling pathways in SW-480 CRC cells. (**A**) Human MAPK phosphorylation array showing the effects of FBL inhibition on signalling pathways in SW-480 cells (**B**) Map of the location of membrane spotted proteins. (**C**) Results of the densitometric quantification indicated that FBL inhibition led to a decrease in the expression of phosphorylated forms of molecules within the MAPK/ERK and PI3K/AKT pathways, except for a slight increase in phosphorylated GSK3β. Phosphorylated molecules within the JNK/p38 pathway were also reduced. Additionally, there was a slight increase in phosphorylated p53 (S15) and HSP27, while phosphorylated CREB showed a marked decrease. Signal intensities from the array were quantified and presented graphically below. Data are expressed as mean ± s.e.m. from two independent experiments. Black and dotted squares indicate the positive and negative controls included in each membrane. Oval form indicates the position of *p*-CREB. (**D**) Western blot analysis of total protein extracts from SW-480 cells was performed to validate the decrease in phosphorylated CREB (*p*-CREB) observed on the array. The results confirmed that while total CREB levels remained unchanged, *p*-CREB levels were significantly reduced upon FBL inhibition, indicating that CREB phosphorylation is dependent on FBL expression (left panel). Quantification of *p*-CREB was normalized to Ku80, with data expressed as mean ± s.e.m. from three independent experiments (right panel). Statistical significance was determined using an unpaired *t*-test, with *p*-values indicated as *p* < 0.05 (*) and *p* < 0.01 (**), *p* < 0.001(***) and *p* < 0.0001 (****), n.s: non-significant (The original Western blot images are in Supplementary Material File S1).

Given that the MAPK pathway is a key signaling cascade downstream of ribosome biogenesis, we selected the human MAPK array (RayBiotech, Inc.^®^, Peachtree Corners, GA, USA) to investigate signaling molecules potentially involved in FBL-mediated migration of SW-480 cells. FBL inhibition reduced the activation of key signaling molecules in the MAPK/ERK, PI3K/AKT, and JNK/p38 pathways, except for a slight increase in phosphorylated GSK3b and Ser15-phosphorylated P53 (Figure 7A,B). Among transcriptional regulators, HSP27 phosphorylation increased, while phosphorylated CREB significantly decreased (Figure 7C). To confirm the role of FBL in regulating CREB activity, we performed a Western blot analysis in SW-480 cells. While total CREB levels remained unchanged, phosphorylated CREB was significantly reduced upon FBL inhibition (Figure 7D), validating the phosphorylation array results. These findings suggest that FBL inhibition is associated with reduced CREB phosphorylation and MAPK pathway activity, which may contribute to the impaired migration of SW-480 cells.

### 3.8. FBL Modulates Tumor Growth in Vivo

**Figure 8:**
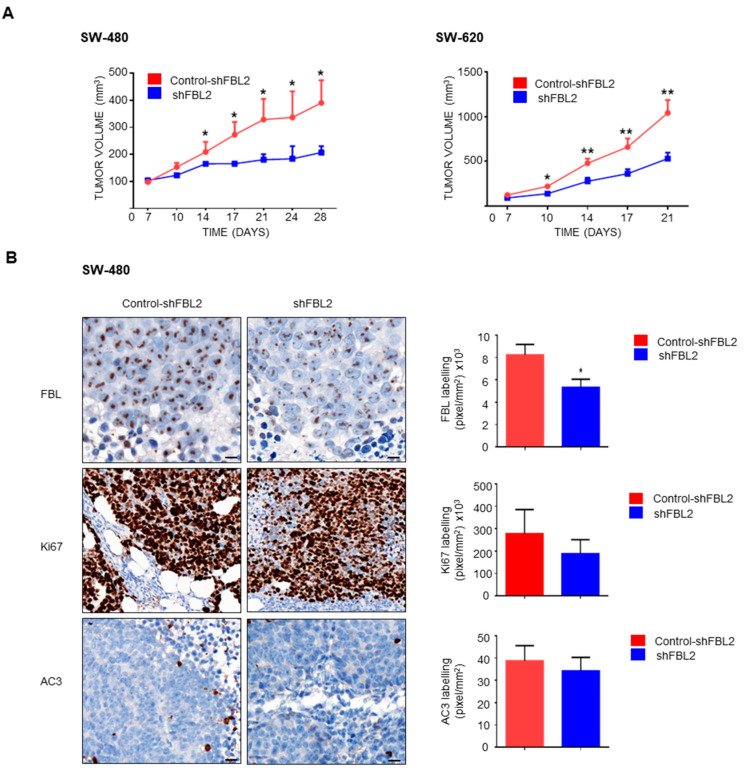
FBL modulates tumour growth in vivo. (**A**) In vivo tumour growth was assessed in SCID mice injected subcutaneously with SW-480 (**left**) and SW-620 (**right**) cell lines (5 × 10^6^ cells) into the flank. Mice were treated with doxycycline (1 mg, i.p.) twice weekly to induce FBL inhibition. Tumours with inhibited FBL expression exhibited significantly slower growth compared to control tumours. Tumour volume was measured over time and is expressed as mean ± s.e.m. from independent experiments (*n* = 5). Statistical significance was determined using unpaired *t*-tests, with *p*-values indicated as *p* < 0.05 (*) and *p* < 0.01 (**). (**B**) Immunohistochemistry (IHC) analysis was conducted on tumour sections from doxycycline-treated and control mice. FBL expression was significantly reduced in the treated group (**top**). A lower Ki-67 index, indicating reduced cellular proliferation, was observed in tumours from the FBL-inhibited SW-480 cells compared to controls (**middle**). No significant difference in activated caspase-3 was detected between treated and untreated groups (**bottom**). Quantification of labelling (pixels/mm^2 ×^ 10^3^) is shown. Scale bar = 10 μm. Data are expressed as mean ± s.e.m. from independent experiments (*n* = 5). Statistical significance was determined using unpaired *t*-tests, with *p*-values indicated as *p* < 0.05 (*).

Given that FBL downregulation alters EMT-related factors and reduces 3D growth in CRC cells, we investigated its role in tumor progression in vivo. SCID mice were subcutaneously injected with SW-480 and SW-620 cells (5 × 10^6^), and FBL inhibition was induced by administering doxycycline (1 mg, i.p.) twice weekly once tumors became palpable. FBL inhibition significantly reduced tumor growth in both SW-480 and SW-620 xenografts (Figure 8A). Immunohistochemistry confirmed decreased FBL protein expression in doxycycline-treated tumors (Figure 8B, top). Additionally, Ki-67 labeling was lower in treated tumors, indicating reduced proliferation, while activated caspase 3 levels remained unchanged (Figure 8B, bottom).

## 4. Discussion

This study provides functional evidence supporting the role of FBL in CRC progression and metastasis, based on analyses in paired isogenic CRC cell lines and patient derived samples. While our findings demonstrate that FBL promotes tumor aggressiveness and resistance to chemotherapy, they do not explore whether FBL expression varies across key molecular CRC subtypes such as MSI-H, MSS, or RAS/BRAF-mutant tumors. This represents a critical avenue for future investigation. Defining whether FBL or ribosome biogenesis factors serve as subtype-specific biomarkers could inform more precise therapeutic strategies. Integrating multi-omic analyses and large-scale public datasets will be essential to determine if FBL-driven pathways are enriched in specific molecular subtypes and to guide the development of targeted interventions. Lymph node metastasis is a key prognostic factor in CRC, driven by EMT, signaling pathways, and tumor microenvironment modulation [23]. FBL was highly upregulated in CRC cell lines, especially in metastatic SW-620 cells, suggesting its role in malignancy. FBL inhibition reduced growth in both lines, with SW-620 affected in 2D and 3D, while SW-480 was impacted only in 3D. In SCID mice, tumor growth was reduced in both, reinforcing the tumor microenvironment’s role, consistent with CRC behavior in 2D vs. 3D environments [24]. In vivo, FBL knockdown significantly impaired tumor growth in subcutaneous xenografts, supporting its functional role in CRC progression. These results align with our in vitro findings in both 2D and 3D models and reinforce the relevance of FBL in sustaining tumorigenicity. However, additional experimental approaches, such as tail vein or orthotopic injection models, are required to assess the role of FBL in metastatic dissemination and colonization. These models would allow a more comprehensive evaluation of the clinical observation that FBL expression is elevated in human liver metastases compared to matched primary colorectal tumors. FBL inhibition also induced morphological changes, particularly in SW-480 cells, making them more compact, with fewer cytoplasmic extensions, reduced adhesion, and decreased migration and invasion. FBL inhibition increased E-cadherin and decreased Vimentin, shifting cells toward a hybrid epithelial state with reduced migration and invasion. This transition was associated with enhanced 5-FU sensitivity, as observed in HCT15 CRC cells [25]. Similarly, in SW-480 cells, FBL inhibition promoted epithelial traits, likely contributing to increased 5-FU sensitivity.

The role of FBL in migration and invasion is unexplored, but a similar function was reported for dyskerin (DKC1), which promotes migration and invasion in HCT116 and DLD1 CRC cells, with high expression linked to CRC metastasis [26]. FBL and DKC1 are both nucleolar proteins involved in ribosome biogenesis and RNA modification, sharing functional networks [27]. Nucleolin (NCL), another related protein, regulates rRNA maturation [28] and plays a key role in cancer progression [29]. NCL also translocates to the cell surface, inducing EMT and migration in HCT116 CRC cells [30]. Additionally, Qi et al. (2017) found that NCL promotes migration and invasion in esophageal squamous cell carcinoma [31].

The role of ribosome biogenesis alterations in CRC progression was previously reviewed in [32]. However, recent studies reveal additional functions of ribosomal proteins in CRC behavior. For example, the ribosome production factor 2 homolog (RPF2) promotes migration and invasion of HCT116 and HT29 CRC cells by inducing EMT and activating the Akt pathway [33]. RPL21 interacts with the lysosome-associated membrane protein 3 (LAMP3) to enhance focal adhesion and metastasis in HCT15 and HCT8 CRC [34]. RPL5 regulates colon cancer proliferation and migration through MAPK/ERK signaling [35]. The ribosomal RNA Processing 12 Homolog (RRP12) stimulates CRC migration and invasion by activating EMT [36]. These findings emphasize the role of ribosomal proteins beyond protein synthesis in CRC progression.

FBL is critical for maintaining nucleolar integrity and plays a pivotal role in cancer biology through its interactions with snoRNAs [37]. The dysregulation of specific snoRNAs, such as SNORDs, has been linked to altered ribosomal function and associated with tumor progression [38]. In Ishikawa and HEC-1B endometrial cancer cells, SNORD60 guides FBL to methylate PIK3CA mRNA, enhancing its translation, activating the PI3K/AKT/mTOR pathway, and promoting tumor growth and survival, thereby functioning as an oncomiR by modulating FBL activity beyond its canonical role in rRNA modification [39]. FBL, as part of the snoRNP complex with NOP56, NOP58, and NHP2L1 (15.5K), is overexpressed in AML, where high NOP56 levels delay remission [40], while NOP56 downregulation inhibits the proliferation of KRAS-mutant (G13D) CRC cells DLD1 and HCT116 [41]. FBL is also known to interact with nucleolar and coiled-body phosphoprotein 1 (NOLC1) upon atypical chemokine receptor 3 (ACKR3) activation, leading to NOLC1 phosphorylation and binding to FBL [42]. This interaction enhances histone H2A methylation, rRNA transcription, and ribosome production, promoting CRC cell growth [42]. Recent findings indicate that FBL plays a previously unrecognized role by regulating both rRNA and mRNA through 2′-O-methylation, enhancing their stability and expression [43]. In cancer cells, elevated FBL correlates with increased 2′-O-methylated mRNAs in key cancer pathways [43], highlighting its involvement in translation control and post-transcriptional regulation. Further studies are needed to investigate how FBL inhibition affects mRNA translation and stability in CRC models. These findings underscore FBL and its snoRNA/protein complex as key regulators of CRC progression, particularly through their emerging roles in RNA stability, which warrant further investigation.

Our study highlights the role of FBL in modulating CRC cell sensitivity to 5-FU. In initially resistant SW-480 cells, FBL inhibition sensitized them to 10 µM 5-FU, enhancing growth arrest and cell death at higher doses (100–400 µM). In 3D spheroids, FBL inhibition amplified 5-FU effects, reducing growth of SW-480 and SW-620 cells and inhibiting migration and invasion. Both cell lines harbor KRAS mutations and overexpress MYC, a potent FBL activator [44]. While FBL inhibition enhances 5-FU sensitivity in our models, we did not assess whether this involves changes in key metabolic enzymes like thymidylate synthase or dihydropyrimidine dehydrogenase. Future studies will investigate whether FBL modulates 5-FU metabolism directly or acts through broader effects on ribosome function and DNA repair. These broader effects are particularly relevant, as CRC progression is closely linked to dysregulated ribosome biogenesis driven by MYC, oncogenic RAS, and ribosomal factors [15,32]. Targeting MYC [45] and FBL [18,46] could provide a therapeutic approach to reduce CRC tumor growth.

Ribosome structure studies further support the link between FBL inhibition and 5-FU sensitivity. Natchiar et al. (2018) identified 72 of 106 predicted 2′-O-methylation sites in human 80S ribosomes, highlighting their role in RNA stability and protein interactions [47]. Since FBL mediates these modifications, its inhibition may disrupt ribosome function. Previously, we reported that 5-FU metabolites integrate into rRNAs, forming fluorinated ribosomes that selectively translate survival-related mRNAs like IGF-1R, promoting 5-FU tolerance in CRC cells [48]. By altering ribosome structure, FBL inhibition could prevent this selective translation, enhancing 5-FU sensitivity. However, this hypothesis, based on [47], requires further validation to confirm the impact of FBL on ribosome integrity and drug response.

Our findings suggest that FBL may contribute to CRC progression through its association with key oncogenic pathways. Specifically, FBL inhibition was accompanied by reduced activation of MAPK/ERK, PI3K/AKT/mTOR, and JNK/p38 pathways, as well as decreased CREB phosphorylation. CREB1 and CREB5 promote CRC progression by enhancing proliferation, migration, and invasion while inhibiting apoptosis [49,50]. High CREB expression correlates with advanced disease, poor survival, and chemotherapy resistance [51,52,53]. While most studies focus on CREB transcriptional regulation, its activity is primarily controlled post-translationally via phosphorylation at Ser133, O-GlcNAc glycosylation, ubiquitination, and acetylation [53,54]. The specific signaling mediators linking FBL to CREB activation remain unknown. Given that FBL plays a key role in ribosome biogenesis, and ribosome production is closely linked to oncogenic pathways, including mTOR signaling, it is plausible that FBL influences CREB activation through these mechanisms [55]. Disruptions in ribosome biogenesis, caused by mutations or chemical inhibitors, generally suppress proliferative pathways and trigger senescence, apoptosis, or autophagy [8,56]. While our data indicate that FBL inhibition is associated with reduced CREB phosphorylation, these findings remain correlative. Further studies are needed to determine whether CREB is a direct downstream target of FBL, to identify the intermediary signaling mechanisms, and to explore the broader functional implications of this potential interaction in CRC.

## 5. Conclusions

In conclusion, our study demonstrates that FBL is significantly upregulated in metastatic CRC cells, driving tumor growth, migration, and chemotherapy resistance. FBL inhibition reduces tumor progression and enhances sensitivity to 5-FU, highlighting its dual role in ribosome biogenesis and cancer aggressiveness. Targeting FBL presents a promising therapeutic strategy to improve CRC treatment outcomes.

## Data Availability

No datasets were generated during the current study.

## Supplementary Materials

The following supporting information can be downloaded at: https://www.mdpi.com/article/10.3390/cancers17243900/s1, File S1: The untrimmed original image of the Western blot. File S2: Supplementary Materials. File S3: Figure S1: FBL expression and its prognostic value in stage IV CRC; Table S1: Key clinical characteristics of the patients analysed; Table S2: Complete datasets of western blot primary antibodies; Table S3: Complete datasets of RT-qPCR primer sequences; Table S4: Complete datasets of immunofluorescence antibodies; Table S5: Complete datasets of the sh-RNA sequences.

## References

[1] Sung H., Ferlay J., Siegel R.L., Laversanne M., Soerjomataram I., Jemal A., Bray F. (2021). Global Cancer Statistics 2020: GLOBOCAN Estimates of Incidence and Mortality Worldwide for 36 Cancers in 185 Countries. CA Cancer J. Clin..

[2] Medici B., Riccò B., Caffari E., Zaniboni S., Salati M., Spallanzani A., Garajovà I., Benatti S., Chiavelli C., Dominici M. (2023). Early Onset Metastatic Colorectal Cancer: Current Insights and Clinical Management of a Rising Condition. Cancers.

[3] Siegel R.L., Wagle N.S., Cercek A., Smith R.A., Jemal A. (2023). Colorectal Cancer Statistics, 2023. CA Cancer J. Clin..

[4] Eng C., Yoshino T., Ruíz-García E., Mostafa N., Cann C.G., O’Brian B., Benny A., Perez R.O., Cremolini C. (2024). Colorectal Cancer. Lancet.

[5] Xie Y.H., Chen Y.X., Fang J.Y. (2020). Comprehensive Review of Targeted Therapy for Colorectal Cancer. Signal Transduct. Target. Ther..

[6] Zhai Z., Yu X., Yang B., Zhang Y., Zhang L., Li X., Sun H. (2017). Colorectal Cancer Heterogeneity and Targeted Therapy: Clinical Implications, Challenges and Solutions for Treatment Resistance. Semin. Cell Dev. Biol..

[7] Ruggero D., Pandolfi P.P. (2003). Does the Ribosome Translate Cancer?.

[8] Pelletier J., Thomas G., Volarevi S. (2018). Ribosome Biogenesis in Cancer: New Players and Therapeutic Avenues. Nat. Rev. Cancer.

[9] Klinge S., Woolford J.L. (2019). Ribosome Assembly Coming into Focus.

[10] Sloan K.E., Warda A.S., Sharma S., Entian K.D., Lafontaine D.L.J., Bohnsack M.T. (2017). Tuning the Ribosome: The Influence of RRNA Modification on Eukaryotic Ribosome Biogenesis and Function. RNA Biol..

[11] Shubina M.Y., Musinova Y.R., Sheval E. (2016). V Nucleolar Methyltransferase Fibrillarin: Evolution of Structure and Functions. Biochemistry.

[12] Shubina M.Y., Musinova Y.R., Sheval E.V. (2018). Proliferation, Cancer, and Aging—Novel Functions of the Nucleolar Methyltransferase Fibrillarin?. Cell Biol. Int..

[13] Marcel V., Ghayad S., Belin S., Therizols G., Morel A.-P.A.P., Solano-Gonzàlez E., Vendrell J., Hacot S., Mertani H., Albaret M. (2013). P53 Acts as a Safeguard of Translational Control by Regulating Fibrillarin and RRNA Methylation in Cancer. Cancer Cell.

[14] Su H., Xu T., Ganapathy S., Shadfan M., Long M., Huang T.H.M., Thompson I., Yuan Z.M. (2014). Elevated SnoRNA Biogenesis Is Essential in Breast Cancer. Oncogene.

[15] Koh C.M., Gurel B., Sutcliffe S., Aryee M.J., Schultz D., Iwata T., Uemura M., Zeller K.I., Anele U., Zheng Q. (2011). Alterations in Nucleolar Structure and Gene Expression Programs in Prostatic Neoplasia Are Driven by the MYC Oncogene. Am. J. Pathol..

[16] Sun X., Gao C., Xu X., Li M., Zhao X., Wang Y., Wang Y., Zhang S., Yan Z., Liu X. (2023). FBL Promotes Cancer Cell Resistance to DNA Damage and BRCA1 Transcription via YBX1. EMBO Rep..

[17] Zhang J., Yang G., Li Q., Xie F. (2021). Increased Fibrillarin Expression Is Associated with Tumor Progression and an Unfavorable Prognosis in Hepatocellular Carcinoma. Oncol. Lett..

[18] Yang L., Zhang Z., Jiang P., Kong D., Yu Z., Shi D., Han Y., Chen E., Zheng W., Sun J. (2024). Phase Separation-Competent FBL Promotes Early Pre-RRNA Processing and Translation in Acute Myeloid Leukaemia. Nat. Cell Biol..

[19] Ma X.D., Xu S.D., Hao S.H., Han K., Chen J.W., Ling H., Chen R.X., Jin X.H., Cao J.H., Lin J.L. (2022). KLF16 Enhances Stress Tolerance of Colorectal Carcinomas by Modulating Nucleolar Homeostasis and Translational Reprogramming. Mol. Ther..

[20] Lu B., Chen X., Liu X., Chen J., Qin H., Chen S., Zhao Y. (2022). C/D Box Small Nucleolar RNA SNORD104 Promotes Endometrial Cancer by Regulating the 2ʹ-O-Methylation of PARP1. J. Transl. Med..

[21] Luo W., Lin S., Huang Y., Zhu K., Zhang F., Lin J., Qin Y., Zhou Z., Wu W., Liu C. (2022). Bioinformatic Analysis and In Vitro and In Vivo Experiments Reveal That Fibrillarin Participates in the Promotion of Lung Metastasis in Hepatocellular Carcinoma. Bioengineering.

[22] Albaret M.A.M.A., Vermot-Desroches C., Paré A., Roca-Martinez J.-X.J.X., Malet L., Esseily J., Gerossier L., Brière J., Pion N., Marcel V. (2018). Externalized Keratin 8: A Target at the Interface of Microenvironment and Intracellular Signaling in Colorectal Cancer Cells. Cancers.

[23] Zhou H., Liu Z., Wang Y., Wen X., Amador E.H., Yuan L., Ran X., Xiong L., Ran Y., Chen W. (2022). Colorectal Liver Metastasis: Molecular Mechanism and Interventional Therapy. Signal Transduct. Target. Ther..

[24] Reidy E., Leonard N.A., Treacy O., Ryan A.E. (2021). A 3D View of Colorectal Cancer Models in Predicting Therapeutic Responses and Resistance. Cancers.

[25] Long S., Wang J., Weng F., Pei Z., Zhou S., Sun G., Xiang D. (2022). ECM1 Regulates the Resistance of Colorectal Cancer to 5-FU Treatment by Modulating Apoptotic Cell Death and Epithelial-Mesenchymal Transition Induction. Front. Pharmacol..

[26] Hou P., Shi P., Jiang T., Yin H., Chu S., Shi M., Bai J., Song J. (2024). DKC1 Enhances Angiogenesis by Promoting HIF-1α Transcription and Facilitates Metastasis in Colorectal Cancer. Br. J. Cancer.

[27] Belli V., Maiello D., Di Lorenzo C., Furia M., Vicidomini R., Turano M. (2023). New Insights into Dyskerin-CypA Interaction: Implications for X-Linked Dyskeratosis Congenita and Beyond. Genes.

[28] Ginisty H., Sicard H., Roger B., Bouvet P. (1999). Structure and Functions of Nucleolin. J. Cell Sci..

[29] Ugrinova I., Petrova M., Chalabi-Dchar M., Bouvet P. (2018). Multifaceted Nucleolin Protein and Its Molecular Partners in Oncogenesis. Adv. Protein Chem. Struct. Biol..

[30] Wu D.M., Zhang P., Liu R.Y., Sang Y.X., Zhou C., Xu G.C., Yang J.L., Tong A.P., Wang C.T. (2014). Phosphorylation and Changes in the Distribution of Nucleolin Promote Tumor Metastasis via the PI3K/Akt Pathway in Colorectal Carcinoma. FEBS Lett..

[31] Qi J., Li H., Liu N., Xing Y., Zhou G., Wu Y., Liu Y., Chen W., Yue J., Han B. (2015). The Implications and Mechanisms of the Extra-Nuclear Nucleolin in the Esophageal Squamous Cell Carcinomas. Med. Oncol..

[32] Nait Slimane S., Marcel V., Fenouil T., Catez F., Saurin J.C., Bouvet P., Diaz J.J., Mertani H.C. (2020). Ribosome Biogenesis Alterations in Colorectal Cancer. Cells.

[33] Li H., Hu X., Cheng C., Lu M., Huang L., Dou H., Zhang Y., Wang T. (2022). Ribosome Production Factor 2 Homolog Promotes Migration and Invasion of Colorectal Cancer Cells by Inducing Epithelial-Mesenchymal Transition via AKT/Gsk-3β Signaling Pathway. Biochem. Biophys. Res. Commun..

[34] Zhu J., Long T., Gao L., Zhong Y., Wang P., Wang X., Li Z., Hu Z. (2023). RPL21 Interacts with LAMP3 to Promote Colorectal Cancer Invasion and Metastasis by Regulating Focal Adhesion Formation. Cell. Mol. Biol. Lett..

[35] Zhang H., Liu J., Dang Q., Wang X., Chen J., Lin X., Yang N., Du J., Shi H., Liu Y. (2022). Ribosomal Protein RPL5 Regulates Colon Cancer Cell Proliferation and Migration through MAPK/ERK Signaling Pathway. BMC Mol. Cell Biol..

[36] An G., Liu Y., Hou Y., Lei Y., Bai J., He L., Liu Y. (2023). RRP12 Suppresses Cell Migration and Invasion in Colorectal Cancer Cell via Regulation of Epithelial-Mesenchymal Transition. J. Gastrointest. Oncol..

[37] Ruggero D. (2012). Revisiting the Nucleolus: From Marker to Dynamic Integrator of Cancer Signaling. Sci. Signal..

[38] Williams G.T., Farzaneh F. (2012). Are SnoRNAs and SnoRNA Host Genes New Players in Cancer?. Nat. Rev. Cancer.

[39] Wu W., Chen X., Liu X., Bao H., Li Q., Xian J., Lu B., Zhao Y., Chen S. (2023). SNORD60 Promotes the Tumorigenesis and Progression of Endometrial Cancer through Binding PIK3CA and Regulating PI3K/AKT/MTOR Signaling Pathway. Mol. Carcinog..

[40] Marcel V., Catez F., Berger C.M., Perrial E., Plesa A., Thomas X., Mattei E., Hayette S., Saintigny P., Bouvet P. (2017). Expression Profiling of Ribosome Biogenesis Factors Reveals Nucleolin as a Novel Potential Marker to Predict Outcome in AML Patients. PLoS ONE.

[41] Yang Z., Liang S.Q., Zhao L., Yang H., Marti T.M., Hegedüs B., Gao Y., Zheng B., Chen C., Wang W. (2022). Metabolic Synthetic Lethality by Targeting NOP56 and MTOR in KRAS-Mutant Lung Cancer. J. Exp. Clin. Cancer Res..

[42] Yang J., Miao R., Li Y., Pan T., Wu S., Qu X., Cui S. (2022). Atypical Chemokine Receptor 3 Induces Colorectal Tumorigenesis in Mice by Promoting β-Arrestin-NOLC1-Fibrillarin-Dependent RRNA Biogenesis. Acta Pharmacol. Sin..

[43] Li Y., Yi Y., Gao X., Wang X., Zhao D., Wang R., Zhang L.S., Gao B., Zhang Y., Zhang L. (2024). 2′-O-Methylation at Internal Sites on MRNA Promotes MRNA Stability. Mol. Cell.

[44] Hu X., Fatima S., Chen M., Huang T., Chen Y.W., Gong R., Wong H.L.X., Yu R., Song L., Kwan H.Y. (2021). Dihydroartemisinin Is Potential Therapeutics for Treating Late-Stage CRC by Targeting the Elevated c-Myc Level. Cell Death Dis..

[45] Whitfield J.R., Soucek L. (2021). The Long Journey to Bring a Myc Inhibitor to the Clinic. J. Cell Biol..

[46] Shi Y., El-Deeb I.M., Masic V., Hartley-Tassell L., Maggioni A., von Itzstein M., Ve T. (2021). Discovery of Cofactor Competitive Inhibitors against the Human Methyltransferase Fibrillarin. Pharmaceuticals.

[47] Natchiar S.K., Myasnikov A.G., Hazemann I., Klaholz B.P. (2018). Visualizing the Role of 2′-OH RRNA Methylations in the Human Ribosome Structure. Biomolecules.

[48] Therizols G., Bash-Imam Z., Panthu B., Machon C., Vincent A., Ripoll J., Nait-Slimane S., Chalabi-Dchar M., Gaucherot A., Garcia M. (2022). Alteration of Ribosome Function upon 5-Fluorouracil Treatment Favors Cancer Cell Drug-Tolerance. Nat. Commun..

[49] Fang Z., Lin A., Chen J., Zhang X., Liu H., Li H., Hu Y., Zhang X., Zhang J., Qiu L. (2016). CREB1 Directly Activates the Transcription of Ribonucleotide Reductase Small Subunit M2 and Promotes the Aggressiveness of Human Colorectal Cancer. Oncotarget.

[50] Li J., Tang Q., Dong W., Wang Y. (2020). CircBACH1/Let-7a-5p Axis Enhances the Proliferation and Metastasis of Colorectal Cancer by Upregulating CREB5 Expression. J. Gastrointest. Oncol..

[51] Wang S., Qiu J., Liu L., Su C., Qi L., Huang C., Chen X., Zhang Y., Ye Y., Ding Y. (2020). CREB5 Promotes Invasiveness and Metastasis in Colorectal Cancer by Directly Activating MET. J. Exp. Clin. Cancer Res..

[52] Ahmed M.B., Alghamdi A.A.A., Islam S.U., Lee J.S., Lee Y.S. (2022). CAMP Signaling in Cancer: A PKA-CREB and EPAC-Centric Approach. Cells.

[53] Steven A., Seliger B. (2016). Control of CREB Expression in Tumors: From Molecular Mechanisms and Signal Transduction Pathways to Therapeutic Target. Oncotarget.

[54] Wen A.Y., Sakamoto K.M., Miller L.S. (2010). The Role of the Transcription Factor CREB in Immune Function. J. Immunol..

[55] Piazzi M., Bavelloni A., Gallo A., Faenza I., Blalock W.L. (2019). Signal Transduction in Ribosome Biogenesis: A Recipe to Avoid Disaster. Int. J. Mol. Sci..

[56] Awad D., Prattes M., Kofler L., Rössler I., Loibl M., Pertl M., Zisser G., Wolinski H., Pertschy B., Bergler H. (2019). Inhibiting Eukaryotic Ribosome Biogenesis. BMC Biol..

