# Supplementary material for: Fibrillarin Contributes to the Oncogenic Characteristics of Colorectal Cancer Cells and Reduces Sensitivity to 5-Fluorouracil
Source: Cancers (Basel). 2025 Dec 5;17(24):3900. doi: 10.3390/cancers17243900 (PMC12730408; doi:10.3390/cancers17243900)

# Western Blot picture for article

# Figure1B

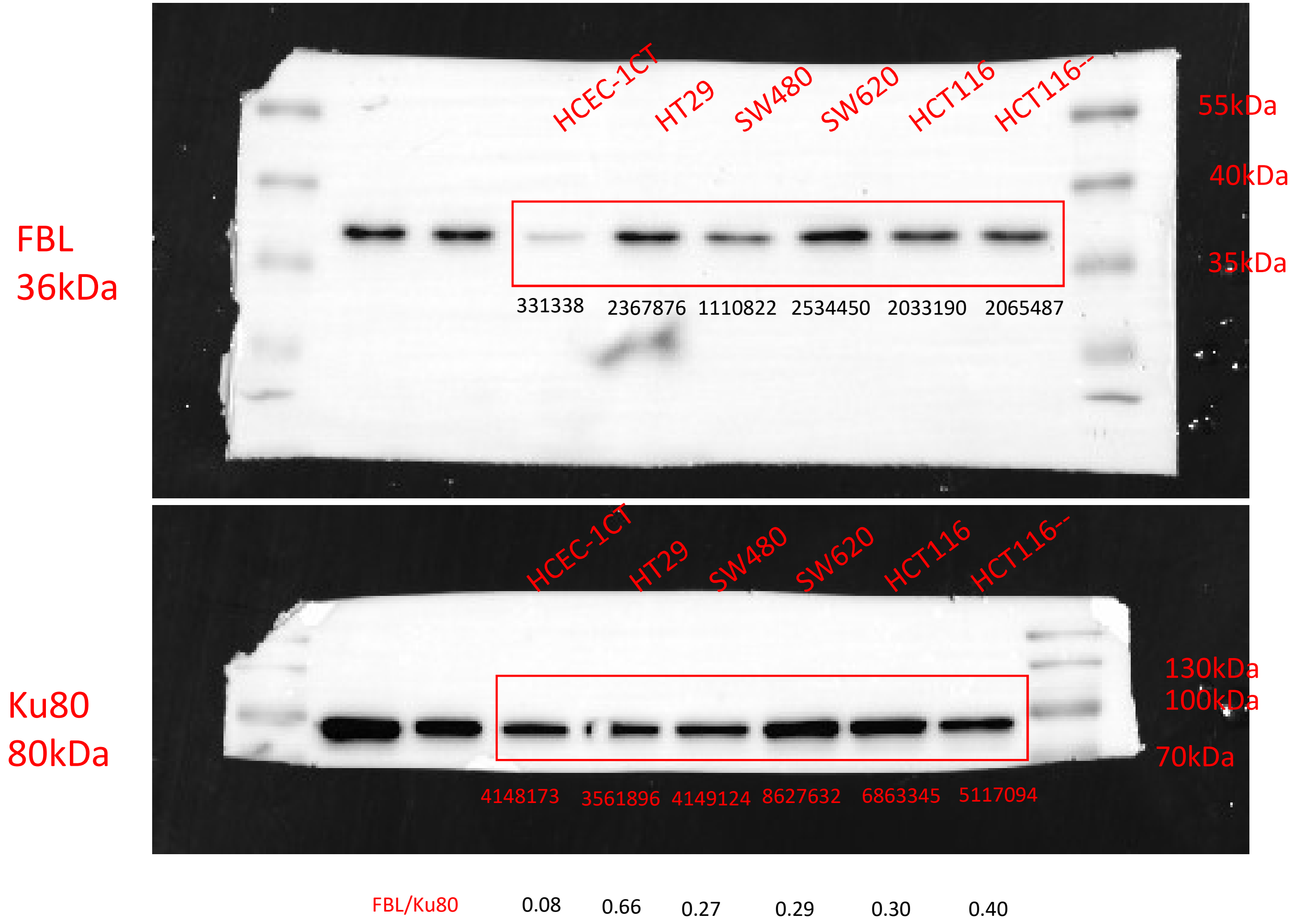


markers cover a spectrum of 10–250 kDa

# Figure2B

markers cover a
spectrum of 10–250 kDa

SW480

SW480

NS- NS+ sh67- sh67+ sh68- sh68+
1 2 3 4 5 6

FBL
36kDa

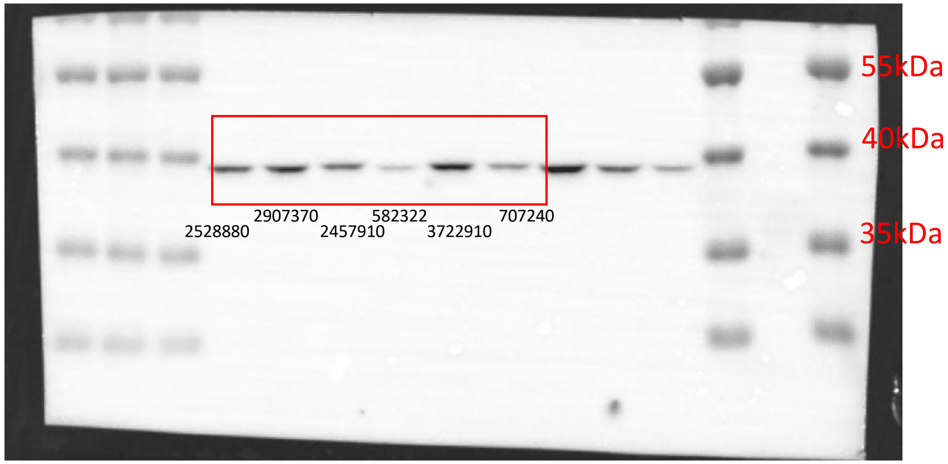

55kDa
40kDa
35kDa

2528880 2907370 2457910 582322 3722910 707240

Ku80
36kDa

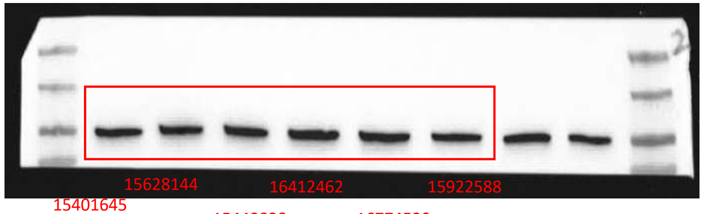

130kDa
100kDa
70kDa

15401645 15628144 15449838 16412462 16774520 15922588

FBL/Ku80 0.16 0.19 0.16 0.04 0.22 0.04

stain free blot

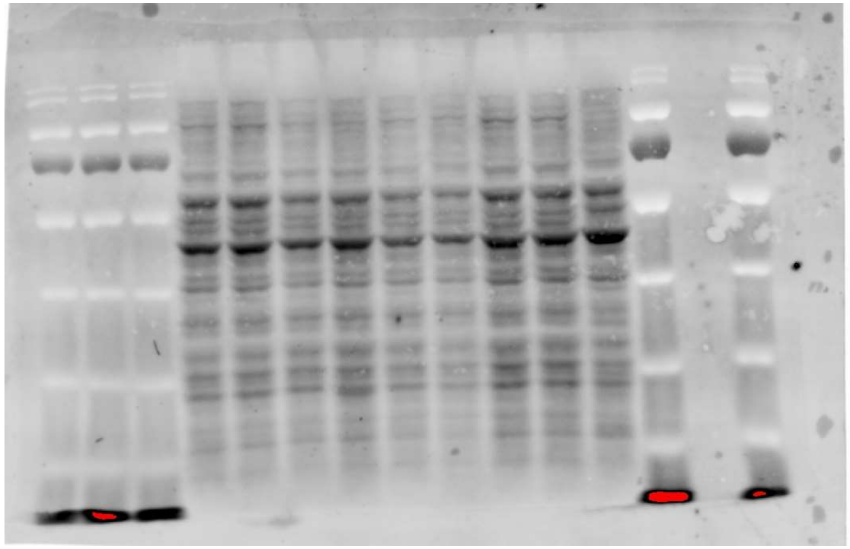

# Figure2D

SW620shFBL

FBL
36kDa

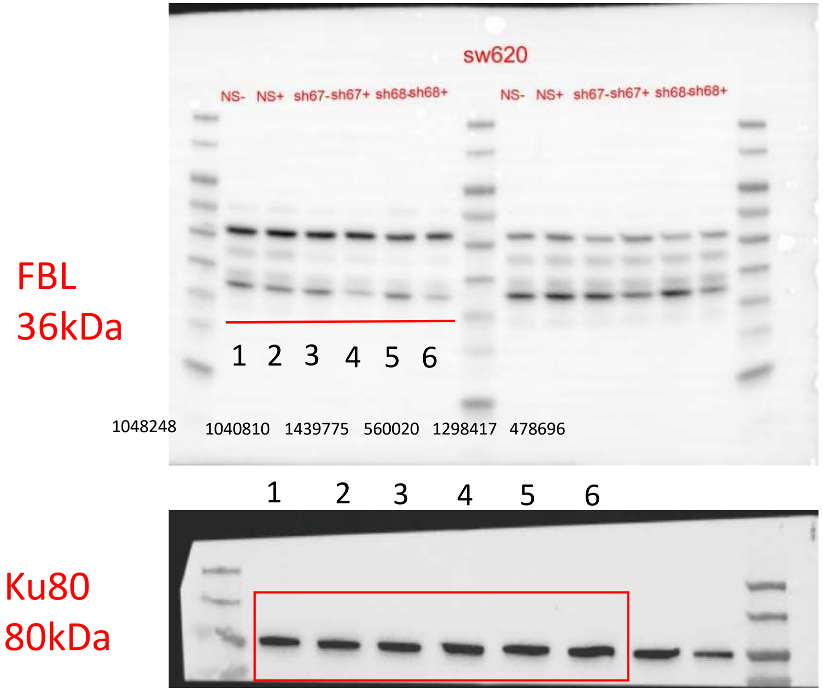


1048248

70kDa
55kDa
40kDa
35kDa

1 2 3 4 5 6

Ku80
80kDa

130kDa
100kDa
70kDa

13492995 14750632 14598450 14009596 14004198 13797795

FBL/Ku80 0.08 0.07 0.10 0.04 0.09 0.03

stain free blot

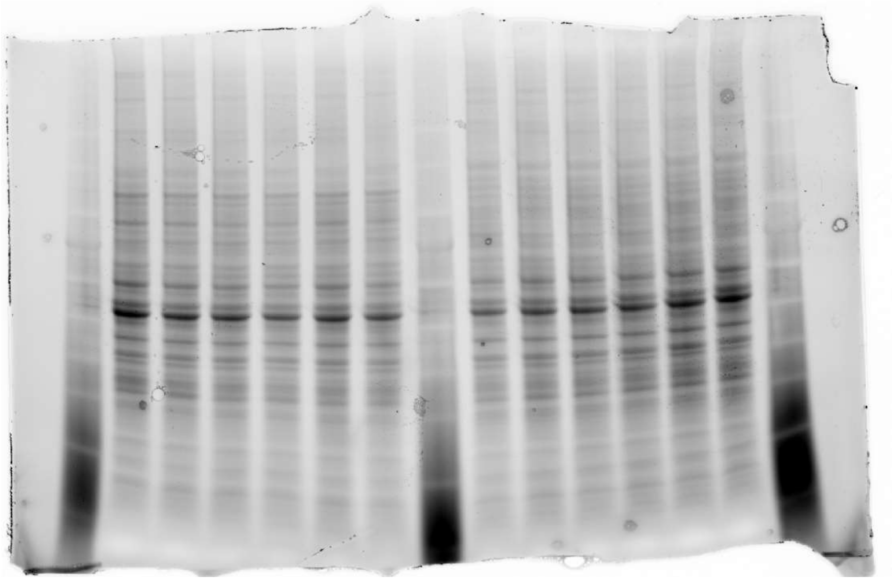

markers cover a
spectrum of 10–250 kDa

# Figure4D

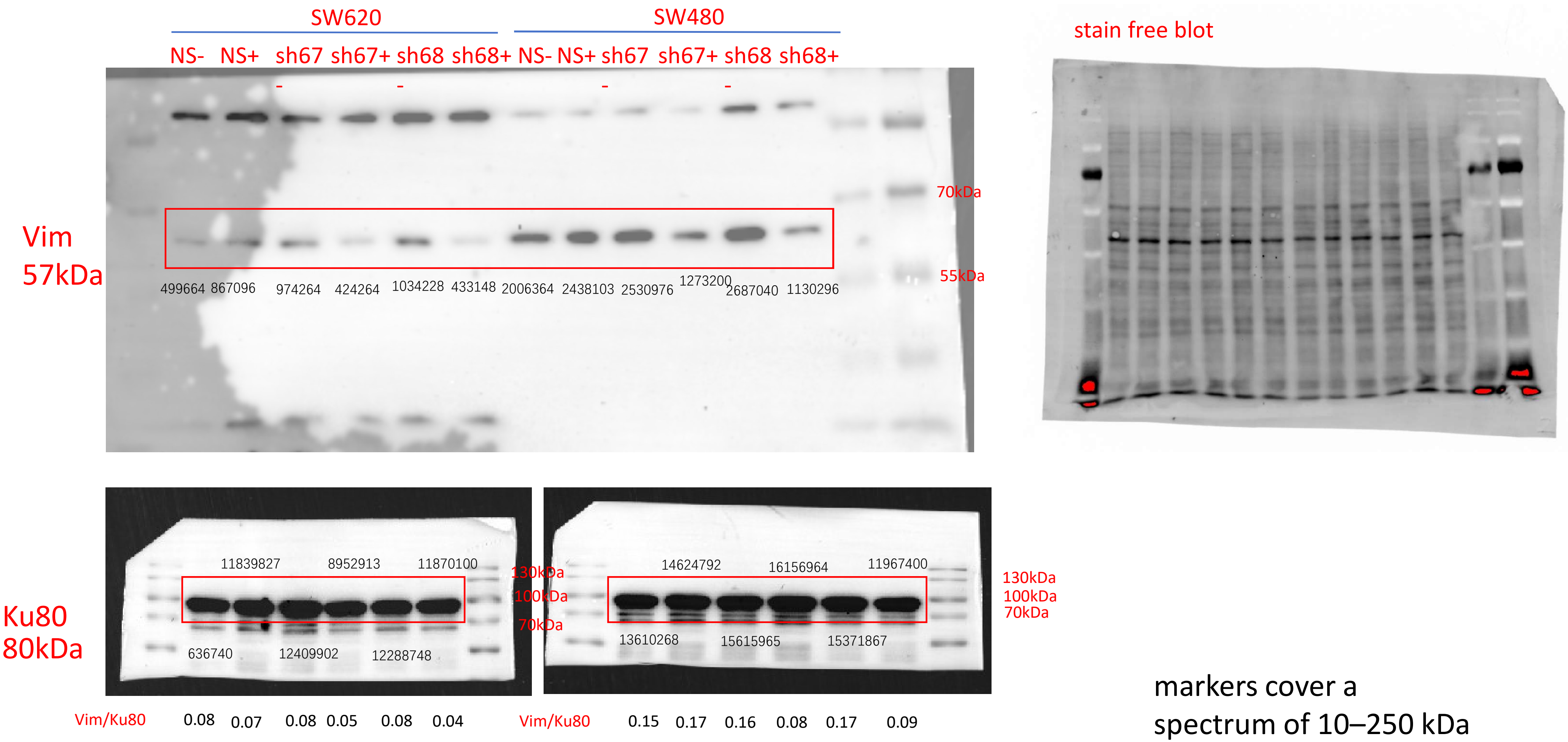


markers cover a spectrum of 10–250 kDa

# Figure4D

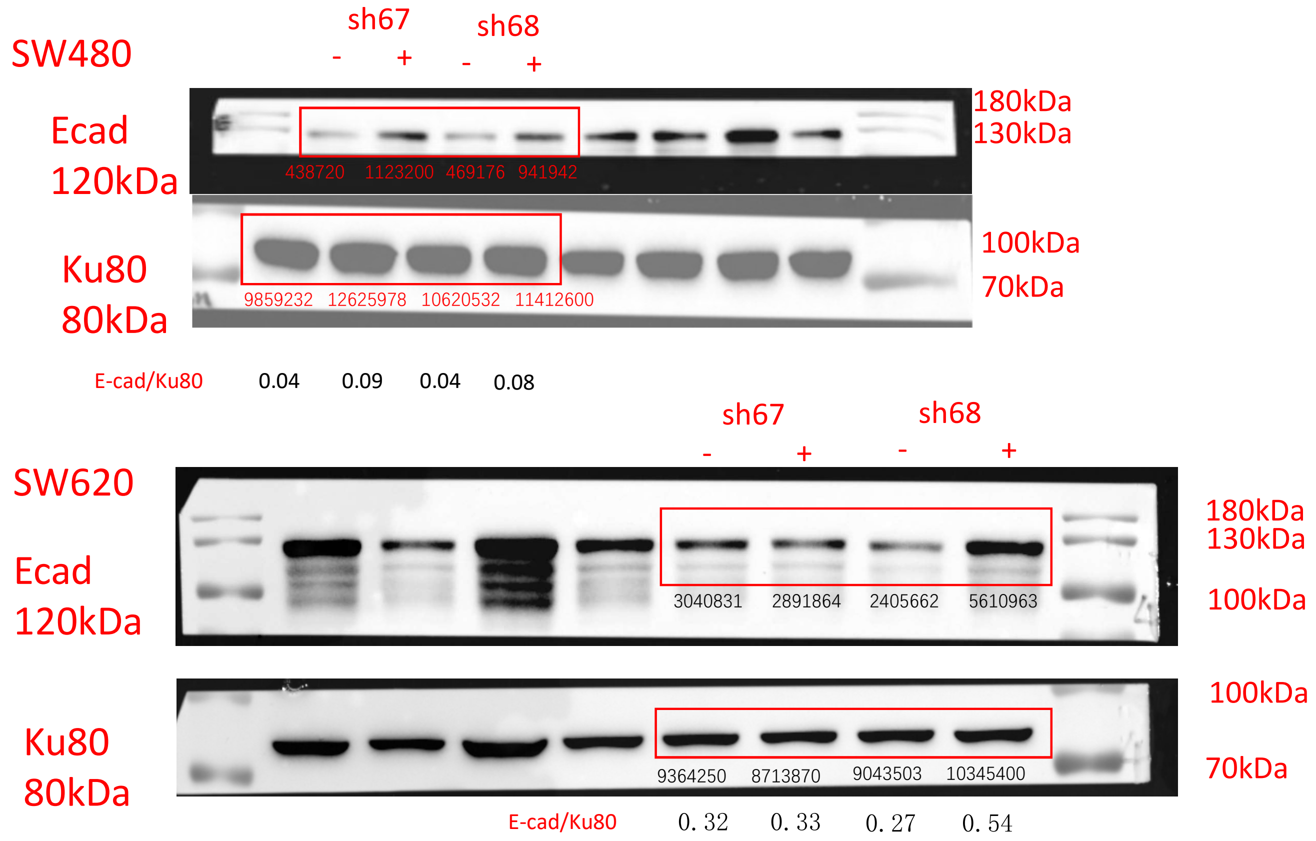


markers cover a spectrum of 10–250 kDa

# Figure7D
p-CREB

markers cover a spectrum of 10–250 kDa

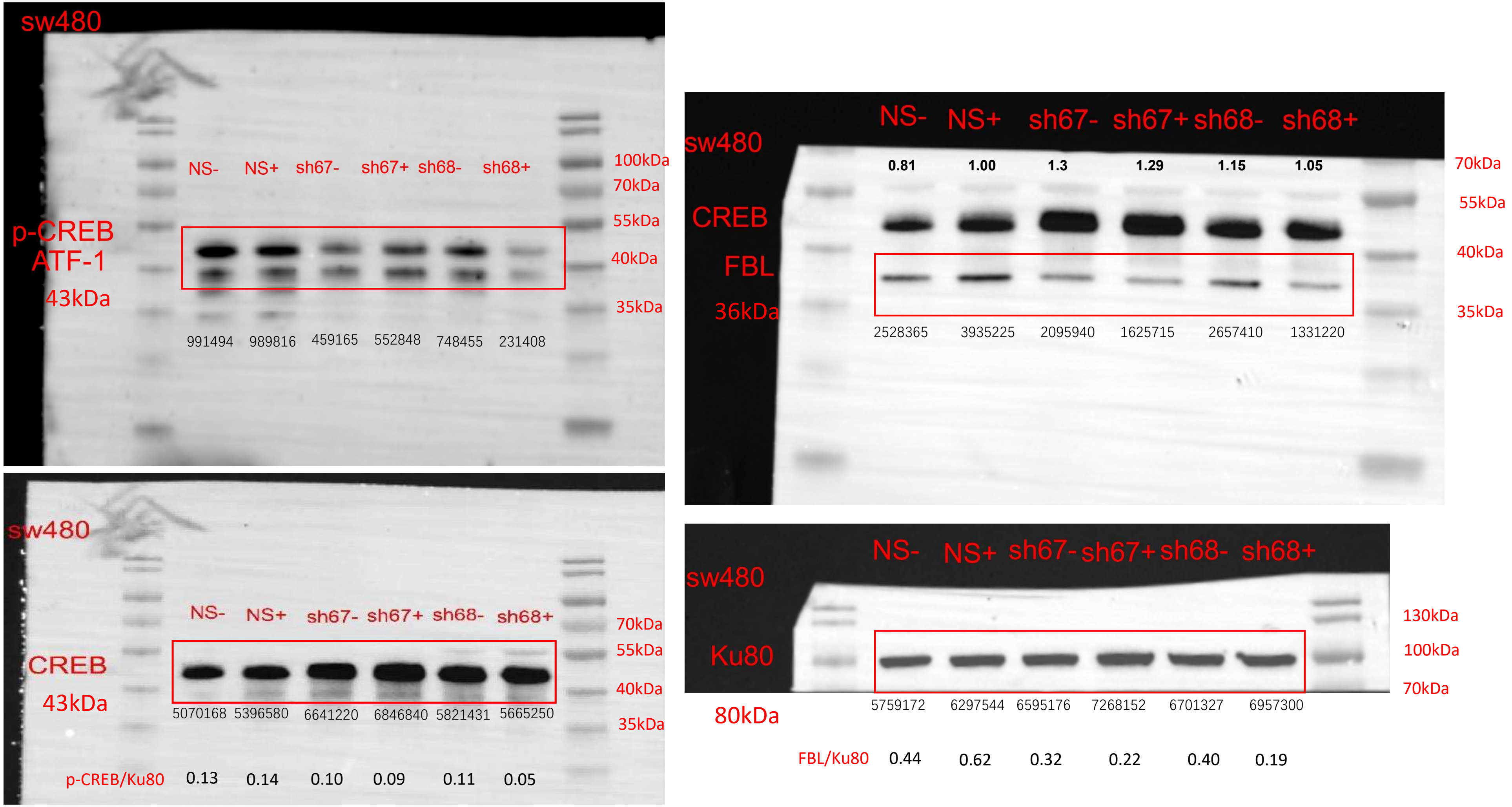

Supplement: Supplementary file 1 [file cancers-17-03900-s001.zip › cancers-3942259-Supplementary-file S1.pdf]
