# Supplementary material for: Fibrillarin Contributes to the Oncogenic Characteristics of Colorectal Cancer Cells and Reduces Sensitivity to 5-Fluorouracil
Source: Cancers (Basel). 2025 Dec 5;17(24):3900. doi: 10.3390/cancers17243900 (PMC12730408; doi:10.3390/cancers17243900)
Supplement: Supplementary file 1 [file cancers-17-03900-s001.zip › cancers-3942259-Supplementary-file S2.pdf]

cancers

Supplementary Materials

Cell culture

Authentication by genotyping of the cell lines was done by GenoScreen (Lille, France). They were cultured in DMEM high glucose 4.5g/L GlutaMAX (Gibco) supplemented with 10% fetal bovine serum (Capricorn Scientific) and 1% penicillin-streptomycin. HEK293T were from ATCC and cultured in DMEM supplemented as described above. All cells were routinely tested against mycoplasma infection. 5-FU was kindly provided by the Centre Léon Bérard (Sanofi-Aventis, FRANCE). The stock solution (384mM) was diluted immediately before use in DMEM.

Protein extraction and western blotting

Cells were lysed in Laemmli buffer (125mM Tris-HCl pH6.8, 2% SDS, 200mM DTE, 20% glycerol) (ThermoScientific, Germany), denatured at 95°C for 10 min, and centrifuged at 13,000 rpm for 15 min. Protein lysates were quantified using 24% TCA. Equal protein amounts were separated via SDS-PAGE (8-12%) and transferred to 0.45µm nitrocellulose membranes (Amersham Protran, 10600003). Membranes were blocked in 1X TBS with 0.05% Tween 20 and 5% non-fat milk, then incubated overnight with primary antibodies (Supplementary Table S1). HRP-conjugated secondary antibodies (Jackson ImmunoResearch) enabled detection by chemiluminescence (ECL, GE Healthcare).

Real-time quantitative PCR analysis

Total RNA was extracted using Trizol reagent (Thermo Fisher Scientific) per the manufacturer's instructions and quantified with a NanoDrop 2000 spectrophotometer (Thermo Scientific). RT was performed on 200 ng of RNA using the PrimeScript RT kit (Takara, RR037B) with 2.5µM oligo dT primers for poly-A RNAs and 5µM random 6-mer primers for non-poly-A RNAs (rRNA, snoRNAs). Real-time qPCR was conducted using LightCycler® 480 SYBR Green I Master Mix (Roche, 4887352001) on an LC96 device or Master Mix 2X EvaGreen (BioRad) on a BioMarkHD device (StarLab), with in-house designed primers (Supplementary Table S2). Relative fold-changes were calculated using the $2^{-\Delta\Delta CT}$ method normalizing to Human XpressRef Universal Total RNA (Qiagen) and GAPDH as housekeeping gene. Each experiment was conducted and analysed in triplicate.

Immunofluorescence

Cells grown on glass coverslips were fixed in 4% of paraformaldehyde in 1XPBS for 10 minutes. Cells were washed in 1XPBS-20mM glycine, permeabilized for 5 minutes in 0.5% Triton X-100 in 1XPBS, washed again and blocked with 3% FBS for 1 h. Primary antibodies (Supplementary Table S4) were applied for 1 h. After washing, cells were incubated with 555 AlexaFluor goat anti -mouse antibody (Invitrogen) or 488 AlexaFluor goat anti -rabbit antibody (Invitrogen) for 1 h. Nuclei were counterstained using Hoescht 33342. Coverslips were mounted using the Fluoromount G mounting medium (EMS). Images were acquired with a Zeiss Wildfield Fluo/HIC Observer microscope and a Zeiss confocal microscope (LSM 880 NLO).

 https://doi.org/10.3390/cancers17243900

Cell Growth, viability, and apoptosis assays

Cell growth, viability, and apoptosis were assessed using multiple methods. Live and dead cells were counted using Trypan blue (Invitrogen, T10282). The IC50 for 5-FU-treated SW-480 and SW-620 CRC cells was determined using the Cell Titer 96 assay (Promega, G3581), and xCELLigence real-time cell analysis (RTCA) system (Agilent Technologies, USA). SW-480 and SW-620 shNS and shFBL cells were pre-treated with 1 µg/mL doxycycline for 4 days before plating (3000 cells/well) in 96-well plates. Viability was assessed over 1 to 6 days by MTS tetrazolium reduction, with absorbance measured at 490 nm (TECAN Spark® multimode microplate reader). Cell growth was monitored in real time using RTCA, which measures electrical impedance every 15 minutes for 6 days. Cells were seeded in E-plates (1×10^4 cells/well) and treated with 5-FU after 24 hours. Incucyte (Sartorius, Incucyte® S3) tracked real-time cell growth and apoptosis. Apoptotic cells were detected using Caspase 3/7 Green or Red Dye. IC50 values for 5-FU were calculated using GraphPad Prism.

Colony formation assay

Cells pre-treated with doxycycline for four days were seeded (1,000 cells/well) in 6-well plates. Media was refreshed weekly with or without doxycycline. After two weeks, colonies were fixed with 4% paraformaldehyde, stained with 1% crystal violet, rinsed, and air-dried. Colonies were photographed, counted, and averaged. Experiments were performed independently at least three times.

Cell migration and invasion assay

SW480-shNS, SW480-shFBL1/2, SW620-shNS, and SW620-shFBL1/2 cells were pre-treated with 1 µg/mL doxycycline for four days. Migration and invasion were analysed using the RTCA system with CIM-plates®, which function as a Boyden chamber. The surface of the upper chamber was spotted with 8 µm pores. The lower chamber contained 10% FCS medium. For invasion assays, the upper chamber was Matrigel-coated (1:10) which left to polymerize at 37°C for 1 hour. Impedance was recorded every 15 minutes for 24-48 hours.

Mice Xenograft model and tumour analysis

Antitumor activity was analysed in immunocompromised models. Six-week-old female CB17 severe combined immunodeficient (SCID) mice purchased from Charles River Laboratories were bred under pathogen-free conditions at the animal facility of our institute. Animals were treated in accordance with the European Union guidelines and French laws for laboratory animal care and use. The animals were kept in conventional housing. Access to food and water was not restricted. This study was approved by the CECCAPP Animal Ethics committee. Continuous health monitoring was carried out on a regular basis, with daily monitoring of clinical symptoms and adverse effects.

Animals of an average weight of 20 g were inoculated subcutaneously (SC) with exponentially growing SW-480 (2.5x106 cells) and SW-620 (5x106 cells) shFBL and shNS cell lines into the right flank in 0.2 mL of PBS. When SC tumours reached a median volume of 100 mm3 animals were randomized and treatments were initiated. The treatment group received intraperitoneal injections of doxycycline (1 mg) twice weekly. Tumour volumes, along with tumour and animal weights, were measured every three days. Mice were sacrificed when tumour volumes reached 1000 mm^3. Tumours were excised, with one half frozen and the other half fixed overnight in 10% formalin. IHC staining was conducted on 4-µm thick sections after heat-induced antigen retrieval (DakoCytomation), using antibodies for FBL (ab166630; Abcam), activated caspase 3, Ki67

(Eurobio, #M3064), Vimentin (Abcam, #92547;), E-cadherin (BD Bioscience, #610182), pCREB (Ser133, Cell Signaling #9198), and CREB (86B10, Cell Signaling #9104). Detection was performed with the ultraView universal DAB detection kit (Roche, Basel, Switzerland). Histological analysis of all samples was conducted by a single blinded pathologist. Staining intensity in the TIFF images was quantified using the Halo® image analysis platform (Centre Léon Bérard) and ImageJ softwares.
