# Supplementary material for: Fibrillarin Contributes to the Oncogenic Characteristics of Colorectal Cancer Cells and Reduces Sensitivity to 5-Fluorouracil
Source: Cancers (Basel). 2025 Dec 5;17(24):3900. doi: 10.3390/cancers17243900 (PMC12730408; doi:10.3390/cancers17243900)
Supplement: Supplementary file 1 [file cancers-17-03900-s001.zip › cancers-3942259-Supplementary-file S3.pdf]

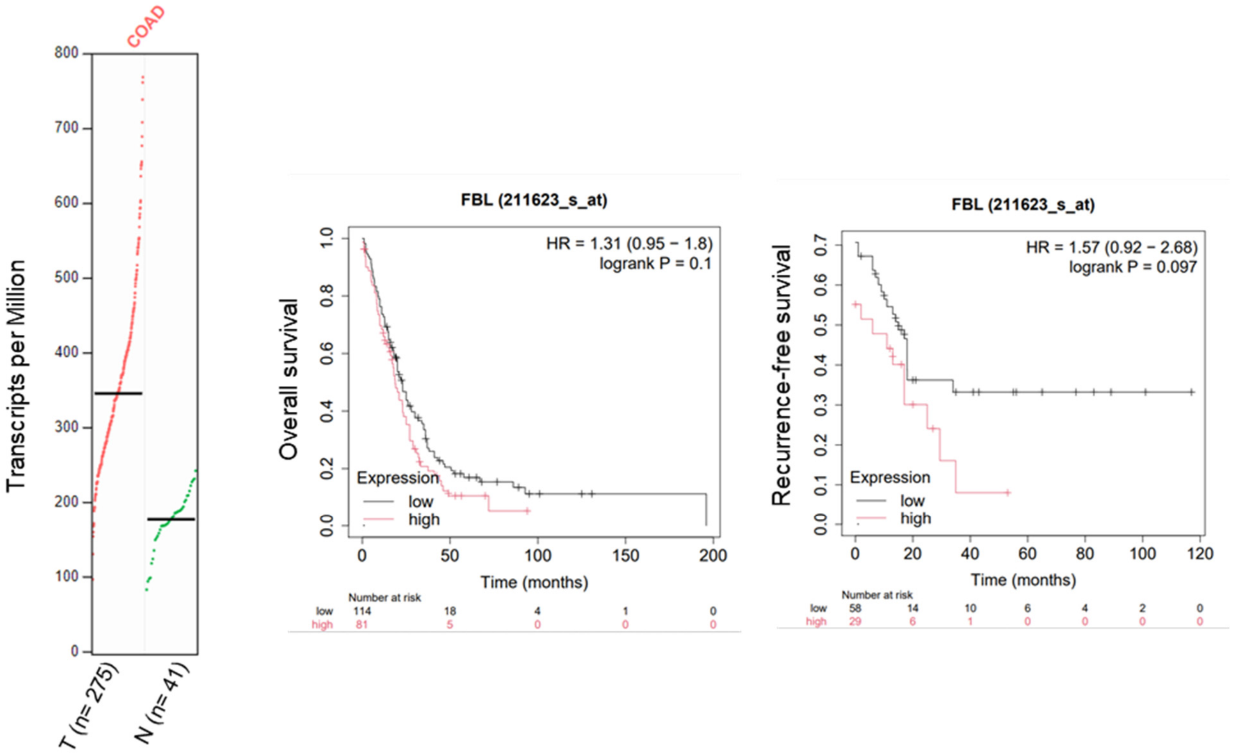

Supplementary Figure S1. FBL expression and its prognostic value in stage IV CRC. GEPIA platform analysis shows significantly higher FBL expression in 275 CRC samples compared to 349 normal colon and rectal tissue samples (left). Kaplan-Meier Plotter analysis indicates that stage IV CRC patients with high FBL expression have significantly lower overall survival and recurrence-free survival than those with low FBL expression (right).

Supplementary Tables

Table S1. Key clinical characteristics of the patients analysed.

**Age**	
Median (SD)	65,17 (9,472)
N	28
Delay between primary and metastasis	
Median (SD)	2,172 (4,665)
N	28
OS (months)	
Median (SD)	30,53 (25,17)
N	28
Sexe	
Male	18(64,29%)
Female	10(35,71%)
TNM Stade	
Stade II	3(10,71%)
Stade III	0(0%)
Stade IV	25(89,29%)

Tumor grade	
Low grade	22(78,57%)
High grade	6(21,43%)
Lymphovascular Invasion	
Present	16(57,14%)
Absent	12(42,86%)
Perineural invasion	
Present	14(50,00%)
Absent	14(50,00%)
Pre sampling treatment	
Present	8(28,57%)
Absent	20(71,43%)

Table S1 (end). Key clinical characteristics of the patients analysed

Apparition of metastasis	
Synchronous	25(89,29%)
Metachronous	3(10,71%)
Metastasis localisation	
Liver	23(82,14%)
Peritoneum	2(7,14%)
Lung	3(10,72%)

Table S2. Complete datasets of western blot primary antibodies.

Gene	Reference, Brand	dilution western blot (1/)
FBL	ab166630, Abcam	1000
CDH1	810181, BD Bioscience	1000
N-Cadherin	610921, BD Bioscience	1000
Vimentin	ab92547, Abcam	5000
KU80	119935, Abcam	2000
p-CREB	(Ser133) # 9198, cell signaling	1000
CREB	(86B10) # 9104, cell signaling	1000
KU80	119935, Abcam	2000
Nop56	sc-133839, Santa	1000
Nop58	ab155969, Abcam	1000
C myc	ab32072, Abcam	1000
P21	sc-6246, Santa	1000
Akt	# 4691S C67E7, cell signaling	1000
Cleaved Caspase-3	# 9664 (Asp175), cell signaling	1000
BCL-XL	# 2764 54H6, cell signaling	1000
GAPDH	AM4300, Invitrogen	1000
puromycin	(12D10), sigma	2000
GM130	ab52649, Abcam	1000
Cytokeratin 8	C5301, Sigma	1000
Na/K ATPase	# 3010, cell signaling	1000

Table S3. Complete datasets of RT-qPCR primer sequences

Gene	Forward (5' to 3')	Reverse (5' to 3')
GAPDH	AGC CAC ATC GCT CAG ACA C	GCC CAA TAC GAC CAA ATC C
qFBL	CCT GGG GAA TCA GTT TAT GG	CCA GGC TCG GTA CTC AAT TTT
qEcad	CCC GGG ACA ACG TTT ATT AC	CGTGGCTCAAGTCAAAGTCC
qVIM	GAC CAG CTA ACC AAC GAC AAA	GAA GCA TCT CCT CCT GCA AT
AKT1	TAT GGC GCT GAG ATT GTG TC	CTT AAT GTG CCC GTC CTT GT
qhZeb1	GTG ACG CAG TCT GGG TGT AA	TTG CAG TTT GGG CAT TCA TA
Zeb2	GCA AAC AAG CCA ATC CCA G	GTT GGG CAC ACT AGC TGG AC
qTwist1	GCA GGG CCG GAG ACC TAG	TGT CCA TTT TCT CCT TCT CTG
qSnail1	CAC TAT GCC GCG CTC TTT C	GCT GGA AGG TAA ACT CTG GAT TAG A
IGFR1	TGA GGA TCA GCG AGA ATG TG	CTG AAT CCG GGC TGT GTA GT
CD44	CCA CGT GGA GAA AAA TGG TC	CGA TGC TCA GAG CTT TCT CC
qP21	ACTCTCAGGGTCGAAAACGG	CGGCGTTTGGAGTGGTAGAA
18S	ATG CGG CGG CGT TAT TC	GCG ACG GGC GGT GTG TA
qOCLN	TGGAGGAGGACTGGATCAGG	TCCTGTAGGCCAGTGTCAAA
Ncad	CCTCCAGAGTTTACTGCCATGAC	GTAGGATCTCCGCCACTGATTC
cMYC	CAGCGACTCTGAGGAGGAAC	TCTGACCTTTTGCCAGGAGC
28S	GAGATTCCCACTGTCCCTACC	CGGAATCAGCGGGGAAAGAA
IGF1	TGG ATG CTC TTC AGT TCG TG	ACT CAT CCA CGA TGC CTG TC
BAX	CCACCAGCTCTGAGCAGATC	TGCTCGATCCTGGATGAAACC
ZO-1	TCACGCAGTTACGAGCAAGT	TGAAGGTATCAGCGGAGGGA
BCL2	CTGGGATGCCTTTGTGGAA	CAGCCAGGAGAAATCAAACAGA

Table S4. Complete datasets of immunofluorescence antibodies.

Gene	Reference, Brand	dilution immunofluorescence (1/)
FBL	ab4566 38F3/ab5821, Abcam	100
CDH1	610181, BD Bioscience	100
Vimentin	ab92547, Abcam	60
Phalloidin		1000
Hoechst	33342, MCE	10000
CD44	560977, BD Bioscience	100
CDH2	610921, BD Bioscience	100
β-catenin	sc-7963, Santa	200
Cytokeratin 8	c5301, Sigma	200
p-CREB	(Ser133) # 9198 , cell signaling	1000
CREB	(86B10) # 9104 , cell signaling	200
Vimentin	347M-1, Sigma	100
E-cadherin	PA5-32178, Thermo Fisher	100
Flag	F1804, Sigma	500

Table S5. Complete datasets of the sh-RNA sequences.

sh-FBL1: TGCATCTTTTTCACTTCGG
sh-FBL2: AGACCATCCGGACCAACGA
sh-NS: GCGATCTCGCTTGGGCGAGAGTAAGTA
